# TKTL1: a new candidate gene in non-obstructive azoospermia

**DOI:** 10.1016/j.rbmo.2025.104895

**Published:** 2025-03-04

**Authors:** Agnieszka Malcher, Mikołaj Smolibowski, Tomasz Stokowy, Hermann Bauer, Alicja Patyk, Piotr Jedrzejczak, Jagoda Kostyk, Zuzanna Graczyk, Rim Ibrahim, Katarzyna Bednarek-Rajewska, Anna Berger, Alexander N Yatsenko, Maciej Kurpisz

**Affiliations:** a Institute of Human Genetics, Polish Academy of Sciences, Poznan, Poland; b IT Division, University of Bergen, Bergen, Norway; c Department of Developmental Genetics, Max Planck Institute for Molecular Genetics, Berlin, Germany; d Center of Obstetrics, Gynecology and Infertility Treatment — Pastelova Clinic, Poznan, Poland; e Department of Cell Biology, Poznan University of Medical Sciences, Poznan, Poland; f Department of Clinical Pathology, Poznan University of Medical Sciences, Poznan, Poland; g Department of Obstetrics and Gynecology and Reproductive Sciences, School of Medicine, University of Pittsburgh, Pittsburgh, PA, USA

**Keywords:** male infertility, azoospermia, NOA, *TKTL1* gene, biomarkers

## Abstract

**Research question:**

What role does *TKTL1* gene serve in human spermatogenesis and does its mutation trigger non-obstructive azoospermia (NOA)?

**Design:**

The genetic background of NOA remains poorly explored. Recent studies using next generation sequencing techniques, however, have uncovered some NOA causative genes, including the *TKTL1* gene. The aim of this study was to define the function of the *TKTL1* gene in human spermatogenesis by inducing its overexpression in human testicular primary (hTP) cells, and verifying these results in male gonad tissue samples collected from patients with NOA determining the genes cluster regulated by *TKTL1*.

**Results:**

Successful overexpression of the *TKTL1* gene was achieved in hTP cells; the *TKTL1* gene expression was significantly higher in genetically modified cells compared with both controls applied. RNA-sequencing was used to select 20 genes in which expression significantly differed in the modified hTP cells overexpressing *TKTL1* gene compared with controls. Male gonad tissue samples collected from patients with NOA harbouring a mutation in the *TKTL1* gene showed a significant downregulation in *HERC5, CSF3, HES1* and *HSPA1B* genes, compared with patients with azoospermia without mutation in the *TKTL1* gene (negative control) as well as control samples with normal spermatogenesis. This was in accordance with results obtained with hTP cells, as the overexpression of the *TKTL1* gene correlated with an increased expression level of the mentioned genes compared with controls.

**Conclusions:**

The *TKTL1* gene is likely involved in the regulation of gametogenesis affecting the proliferation and differentiation of germ cells at the early stage of spermatogenesis.

## INTRODUCTION

Non-obstructive azoospermia (NOA) is one of the most severe types of male infertility. It is caused by spermatogenesis dysfunction, leading to cell differentiation arrest (Sharma and Leslie, 2022). It may stem from developmental abnormalities, testicular damage (congenital or secondary), sperm transport disorders or secondary to hormonal imbalances, such as hypogonadism, leading to delays in the initiation of puberty, but notably underlined by genetic causes (Wong et al., 2023).

Despite ongoing research into the aetiopathogenesis of male infertility, knowledge of the genetic background of NOA is insufficient (Ozturk, 2023). Recent studies using next generation sequencing techniques, i.e. whole genome sequencing or whole exome sequencing, were promising as they allowed the identification of novel genes associated with NOA (Krausz et al., 2020; Salas-Huetos et al., 2021; Ghieh et al., 2022; Kherraf et al., 2022; Malcher et al., 2022).

The absence of a good research model that fully overlaps with male spermatogenesis makes it significantly more challenging to indicate precisely the role of relevant genes for this process (Jiang et al., 2022). The current models applied to disclose gene function in humans are mostly based on rodents and do not fully align with some stages of germ cell differentiation. To understand the developmental stages, genes, or both, involved in spermatogenesis and male fertility, animal models, such as *Mus musculus* (Jiao et al., 2021), *Drosophila melanogaster* (Siddall and Hime, 2017) and *Danio rerio* (Hoo et al., 2016) were predominantly used. Not all genes, however, can be considered homologous in animal models either because of distinctive functions they may have in different species, or because of their absence in primates (Jiao et al., 2021). This scenario was found in *Tktl1*-null knockout mice, which showed no reproductive defects or effect on fertilization (Miyata et al., 2016), whereas, in humans, *TKTL1* mutations may affect spermatogenesis (Malcher et al., 2022; Khan et al., 2023).

It is, therefore, essential to find more suitable models to study the process of human spermatogenesis and male fertility. To meet these expectations, cell cultures have been widely used as in-vitro research models. These models have allowed the mechanisms of action and intercellular interactions among male germ cells during their development, proliferation and differentiation to be explored (Saitou and Hayashi, 2021). Active research has also been carried out on Leydig cells, Sertoli cells, spermatogonial stem cells, primordial germ cells and germ cell-like cells derived from induced pluripotent stem cells (Zhu et al., 2012; Cormier et al., 2017; Aydos et al., 2019; Murdock et al., 2019). In-vitro models, together with next generation sequencing techniques, e.g. whole genome or exome sequencing, and targeted genome editing techniques, including CRISPR-CAS9, contribute to understanding the functions of newly discovered genes, especially those that do not have homologues in animal models. Such research may also provide more detailed information about the process of human spermatogenesis and the genetic factors influencing male infertility.

In a previously published study by our group using whole-genome sequencing (Malcher et al., 2022), two novel ultrarare disease-causing variants were identified: chrX:153537712_153537712delG/c.268_268delG/p.Asp90Metfs*35 and *chrX:153556287A*>*G/c.1601A*>*G/p. Glu534Gly* (according to GRCh37), in the *TKTL1* gene (the gene encoding transketolase-like protein 1) in two out of 39 patients with NOA with arrest at the initial stage of spermatogenesis. Both these patients revealed a lack of the *TKTL1* gene expression at mRNA and protein levels. The first variant resulted in the mutation of a negatively charged aspartic acid residue to a hydrophobic amino acid and truncated most of the protein, whereas the second variant, c.1601A>G, led to the amino acid change p.Glu534Gly in the C-terminal domain, suggesting destabilization of the folding in this domain (Malcher et al., 2022).

The *TKTL1* gene is located on the long arm of the X chromosome (Xq28) and encodes the enzyme transketolase 1. Four isoforms of this gene exist, and their products range in mass from 92 kDa down to 25 kDa (Hartmannsberger et al., 2011). *TKTL1* gene expression was previously correlated to different types of cancer, in which enhanced proliferation was observed (Deshpande et al., 2019). Even though the function of *TKTL1* in spermatogenesis remains unclear, its exclusive expression in the testes (*Human Protein Atlas 2025;* ENSG00000007350-TKTL1), and its correlation with proliferation in malignancies, suggests that this gene may play an important role in growth and cell differentiation.

In the present study, the function of the *TKTL1* gene in human spermatogenesis was partially explored by inducing its overexpression in human testicular primary cells, and identifying, to some extent, the gene network regulated by *TKTL1* and verifying it in patients with NOA with or without mutation in *TKTL1*. This may help to predict what and how the variants of this gene could be causative for NOA.

## MATERIALS AND METHODS

### Participants

In the present study, experiments were conducted using commercially available human testicular primary cells (hTP) cells (catalogue number 19105) (Celprogen, Torrance, CA, USA), which were obtained from a 25-year-old white man with normal karyotype (46,XY) and normal spermatogenesis. To characterize the hTP cells (*n* = 3 experimental replicates), the commercial total RNA obtained from normal testicular tissues with normal spermatogenesis was used (*n* = 3) (catalogue number 636533) (Clontech Laboratories, Mountain View, CA, USA); (catalogue number 540049) (Agilent Life Technologies, Carlsbad, CA, USA) (catalogue number AM7972) (Ambion Life Technologies, Carlsbad, CA, USA) and also TCam-2 cells (*n* = 3 experimental replicates) (the testicular seminoma cell line), which were gifted by Dr Sohei Kitazawa (Ehime University Graduate School of Medicine, Japan) and described in detail by Malcher et al. (2023).

For real-time polymerase chain reaction (PCR) verification, the following was used: total RNA extracted from the testicular tissue fragment of a patient with NOA (meiotic arrest) and with *c.1601A*>*G* mutation in the *TKTL1* gene (marked as 34L) (Malcher et al., 2022), and the testicular RNA sample from a patient with NOA (mostly with spermatocytes arrest and with less than five spermatozoa per tubule and only few late spermatids) with no mutation in *TKTL1* gene and compared them with the commercially available RNA obtained from normal testicular tissue with normal spermatogenesis pooled from 39 white participants aged between 16 and 64 years (Clontech Laboratories, Mountain View, CA, USA). For the immunofluorescence staining, commercial human adult testes paraffin sections were used (HP-401) (Zyagen, San Diego, CA, USA).

**FIGURE 1:**
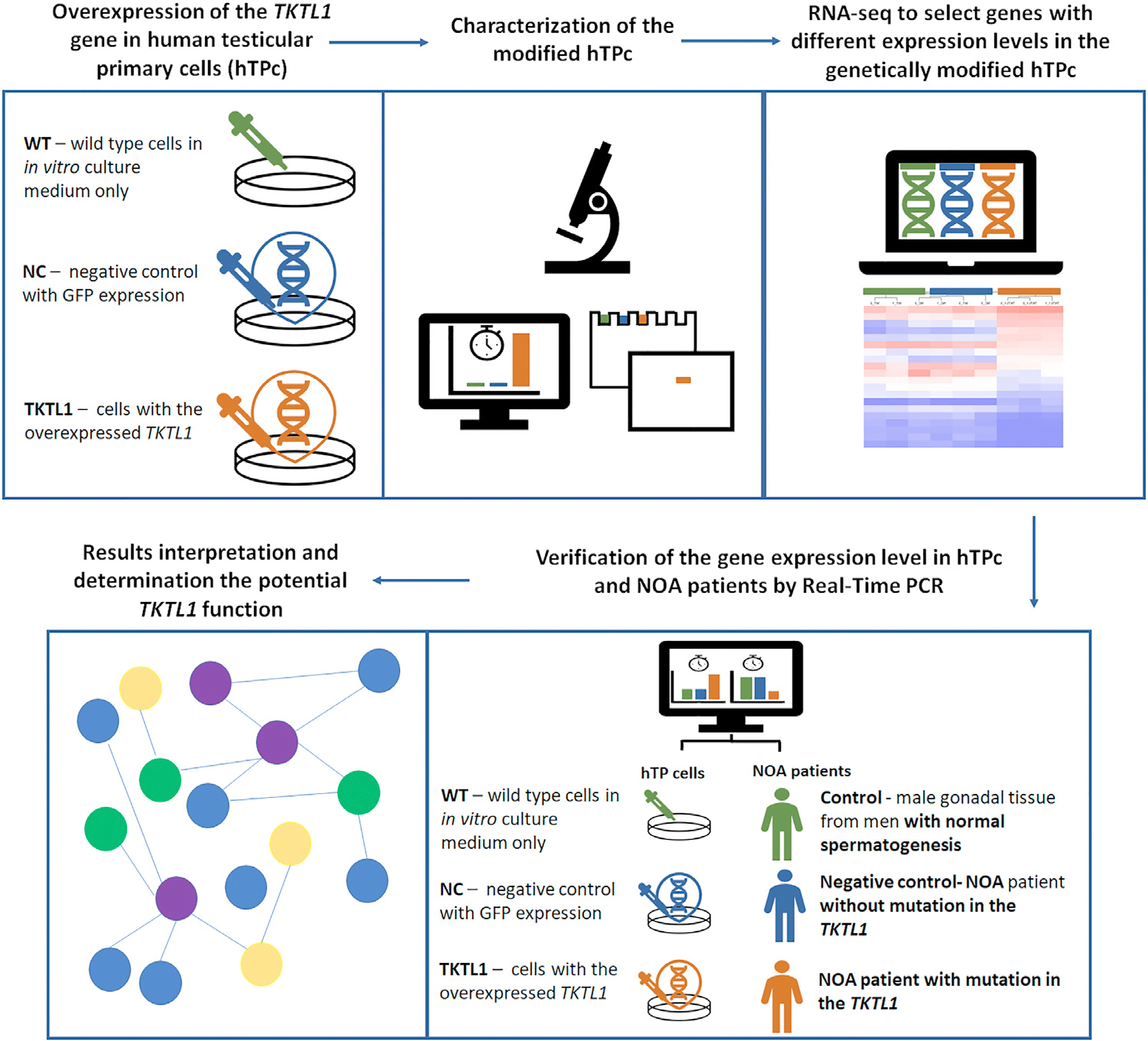
The different study groups and main steps of the study design. Control, male gonadal tissue from men with normal spermatogenesis; hTPC, human testicular primary cells; NC, negative control with GFP (green fluorescent protein) expression; NOA, non-obstructive azoospermia; negative control NOA, patient with NOA and no mutation in the *TKTL1* gene; NOA patient with mutation in the *TKTL1*, patient with NOA with mutation in the *TKTL1* gene; TKTL1, cells with the overexpressed *TKTL1* gene; WT, wild type cells in in-vitro culture medium only.

All experiments were conducted in accordance with the relevant guidelines and regulations. All the experimental protocols used in this study were approved by the Ministry of Climate and Environment, Republic of Poland (permission number 145/2020 and 126/2021) and by the Local Bioethics Committee of Poznan University of Medical Sciences (permission number 1003/18, approval date 11 October 2018), and all participants provided informed consent. The main steps of the study design are presented in FIGURE 1.

### Culture of human testicular primary cell suspension

The hTP cells were cultured on pre-coated flasks with complete extra-cellular matrix (Celprogen, Torrance, CA, USA) and complete media containing serum (Celprogen, Torrance, CA, USA), both designed for human testicular primary cell in-vitro culture. The culture was conducted according to the specified manufacturer’s protocol for human testicular primary cell in-vitro culture (Celprogen, Torrance, CA, USA) in standard conditions at 37°C, 5% CO_2_ and 95% humidity.

### Activation of TKTL1 in TCam-2 cells using CRISPR system

The transfection was conducted in accordance with the procedure previously published by Malcher et al. (2023) using three different px335_G2P vectors with cloned gRNAs specific for the *TKTL1* gene and the PB-TRE-dCas9-VPR vector. The gRNA sequences are listed in the 5′–3′ direction: gRNA_1-TGACTTGCTTGCGGAGGGAG; gRNA_2-GAGTGGCACGAGGCATGCGG and gRNA_3-GAAACGCACTGGGCAGCTCGC. The vector pX335_G2P was a kind gift from Boris Greber (Max Planck Institute for Molecular Biomedicine, Münster) to the Department Herrmann at the Max Planck Institute for Molecular Genetics in Berlin. PB-TRE-dCas9-VPR (Addgene number 63,800) (Addgene, Watertown, MA, USA) was purchased by the Max Planck Institute for Molecular Genetics in Berlin (Department Herrmann), which has the right to use the construct. The cells were selected with puro and doxycycline for 72 h. After 72 h, the cells were collected, and *TKTL1* gene expression was analysed. The experiment was carried out in three experimental replicates: wild type (*n* =3 experimental replicates), negative control with non-specific sgRNAs for the human genome (*n* = 3 experimental replicates) and TKTL1-cells with the activated *TKTL1* gene using specific sgRNAs for the *TKTL1* sequence (*n* = 3 experimental replicates). The transfection efficiency of the modified TCam-2 cells was confirmed in a Juli FL fluorescence microscope (NanoEnTek, Seoul, South Korea), and the percentage of positively transfected cells (green fluorescent protein [GFP]-positive cells) (*n* = 250 counted) was counted with multi-point tool using ImageJ2 (Rueden et al., 2017).

### Generation of *TKTL1*-modified human testicular primary cells

#### Lentiviral plasmid construction

*TKTL1* vector (pLV[Exp]-EF1A>hTKTL1-hPGK>EGFP:T2A:Puro vector) as well as vector with only GFP sequence were used as negative control (pLV[Exp]-Puro-EF1A>EGFP/3xFLAG) and produced by Vector Builder (VectorBuilder Inc., Chicago, IL, USA).

#### Lentiviral packaging

The second-generation packaging system was used for lentiviral particles packing. Plasmids pLV[Exp]-EF1A>hTKTL1-hPGK>EGFP:T2A:Puro or pLV[Exp]-Puro-EF1A>EGFP/3xFLAG, psPAX (12260 Addgene) and MD2G (12259 Addgene) (4:3:1) were mixed, and transfection of the HEK cells (27 × 10^6^) was carried out using a calcium phosphate protocol (Jordan et al., 1996). Pseudoviral particles were collected at 48 and 72 h after transfection, filtered through a 45-*μ*m filter (Millipore, Temecula, CA, USA) and centrifuged using filter units (Amicon Ultra-15) (Merck, Temecula, CA, USA). The aliquots were snap-frozen and stored at −80°C.

#### Determination of the number of virus copies

First, the NucleoSpin RNA Virus Kit (Takara Bio USA, Mountain View, CA, USA) was used to isolate viral RNA. Copy number determination was conducted in accordance with guidelines provided by the manufacturer of the Lenti-X-qRT-PCR Titration Kit (Takara Bio USA, Mountain View, CA, USA). The copy number of virus consisting of pLV[Exp]-EF1A>hTKTL1-hPGK>EGFP:T2A:Puro; psPAX and MD2G was 1,89*10^10^/ml, whereas, for virus consisting of pLV[Exp]-Puro-EF1A>EGFP/3xFLAG, psPAX and MD2G it was 1,11*10^11^/ml.

#### The human testicular primary cells transduction

The hTP cells were transferred into a six-well plate pre-coated with complete extra-cellular matrix dedicated for hTP cells (Celprogen, Torrance, CA, USA). When the cell confluence reached 70–80%, the cells were transduced. For this purpose, a transduction mixture consisting of 500 *μ*l virus of lentiviral vectors with cDNA for the *TKTL1* gene was used. As negative control, the hTP cells were transduced with mixture consisting of 500 *μ*l virus of lentiviral vector with enhanced GFP sequence. The polybrene reagent was also added to the virus mixture in the amount of 10 *μ*l of reagent per 1 ml of the administered to the cells mixture. The transduction mix was supplemented up to 2 ml volume using complete medium dedicated to all culture. After preparation, the transduction mixture was applied to the cells. The cells were then cultured at 37°C in an atmosphere with 5% CO_2_. After 24 h, the medium was collected from the cells and the transduction mixture with the same composition as described above, prepared again and applied to the cells. On the third day, the transduction mixture was replaced with a complete medium dedicated to in-vitro cultured cells. On the fourth day, cell selection with antibiotics was started using puromycin in a concentration of 1 *μ*g/ml diluted in complete medium. The antibiotic selection was continued for 7 days. When the cells reached around 90% confluency, the cells were transferred into larger culture flasks pre-coated with complete extra-cellular matrix dedicated to hTP cells (Celprogen, Torrance, CA, USA). After reaching a confluence (80–90%), the cells were harvested and collected for further analysis. The experiment was conducted in three experimental replicates: wild type cells in in-vitro culture medium only (*n* =3 experimental replicates); negative control with GFP expression (*n* = 3 experimental replicates) and *TKTL1* - cells with overexpression of *TKTL1* gene (*n* =3 experimental replicates). The transduction efficiency of the modified hTP cells was confirmed in a Juli FL fluorescence microscope (NanoEnTek, Seoul, South Korea), and the percentage of positively transfected cells (GFP-positive cells) (*n* = 150 counted) was counted with multi-point tool using ImageJ2 (Rueden et al., 2017).

### RNA and protein extraction

The hTP cells were lysed in 350 *μ*l of RLT buffer with *β*-mercaptoethanol, and RNA and protein were extracted according to the manufacturer’s protocol of the AllPrep DNA/RNA/Protein Mini Kit (Qiagen, Hilden, Germany). In contrast, testicular biopsy specimens (3–5 mm^3^), previously collected in RNAlater solution (Ambion Life Technologies, Carlsbad, CA, USA) during a standard infertility work-up that included histopathological evaluation, were homogenized three times for 20 seconds each at 6 m/s, with 10-second intervals using a BeadBlaster^™^24 (Benchmark Scientific, Sayreville, NJ, USA) in 600 *μ*l of RLT buffer with *β*-mercaptoethanol, and processed following the same manufacturer’s protocol. The RNA samples were additionally purified according to the manufacturer’s protocol of the Turbo DNA-free Kit (Thermo Fisher Scientific, Waltham, MT, USA). The protein was dissolved in a buffer consisting of 8 M urea, 50 mM Tris–HCl, pH 8.0, 1% SDS (1:1) and a protease inhibitor cocktail (Roche, Basel, Switzerland).

### Real-time polymerase chain reaction

The cDNA was synthesized from 1 *μ*g of total RNA using iScript^™^ Reverse Transcription Supermix (Bio-Rad Laboratories, Hercules, CA, USA) in a 20 *μ*l reaction. The real-time PCR was conducted using SsoAdvanced ^™^ SYBR ^®^ Green Supermix (Bio-Rad Laboratories, Hercules, CA, USA) and specific primers for the genes studied (Supplementary Table 1) according to the manufacturer’s protocol. The threshold cycle values of each transcript studied were analysed with a CFX Opus Real-Time PCR Systems (Bio-Rad Laboratories, Hercules, CA, USA) using standard cycling parameters. All the hTP cell samples were run in duplicate: wild type (*n* = 3 experimental replicates), negative control (*n* = 3 experimental replicates), TKTL1 (*n* = 3 experimental replicates) as well as the standard curve were run in duplicate. Expression analysis of the *CALB2, CD68, CYP11A1, DAZL, DDX4, DSG2, FGFR3, MKI67, SOX9, SOX17, SPOCD1* and *UTF1* genes was also conducted in the unmodified hTP cells compared with adult testicular tissue with normal spermatogenesis and unmodified Tcam-2 cells (Supplementary Figure 1). The testicular tissue samples of a patient with NOA without mutation in *TKTL1* (*n* =2 technical replicates) and a patient with NOA with *c.1601A*>*G* mutation in the *TKTL1* gene (*n* = 2 technical replicates) as well as RNA obtained from normal testicular tissue with normal spermatogenesis pooled from 39 patients of white ethnicity (*n* = 2 technical replicates) were run in duplicate. The relative expression level of each studied transcript was normalized with reference to two housekeeping genes (*β-actin* and *GAPDH*) according to CFX Maestro software (Bio-Rad Laboratories, Hercules, CA, USA).

### Western immunoblotting

The 50 *μ*g of protein from GTP cells (wild type: *n* = 3 experimental replicates; negative control: *n* = 3 experimental replicates; and TKTL1: *n* = 3 experimental replicates) was separated on 4–20% Mini-PROTEAN^®^ TGX Stain-Free^™^ Protein Gels (Bio-Rad Laboratories, Hercules, CA, USA), electrotransferred in mix condition (7′ ) using Trans-Blot^®^ Turbo (Bio-Rad Laboratories, Hercules, CA, USA) to PVDF membrane (Bio-Rad Laboratories, Hercules, CA, USA). The membrane was blocked with buffer containing non-fat milk (Bio-Rad Laboratories, Hercules, CA, USA). Immunodetection was carried out using the following antibodies: primary antibodies: anti-TKTL1 mouse (catalogue number SAB1409857) (Sigma-Aldrich, St Louis, MO, USA) 1:250–65 kDa; anti-GAPDH rabbit (catalogue number ab9485) (Abcam, Cambridge, UK) 1:1000–37 kDa; secondary antibodies: anti-mouse (RRID: AB_258476) (Sigma-Aldrich, St Louis, MO, USA) 1:10,000; anti-rabbit (AB_10679369) (Abcam, Cambridge, UK) 1:40,000. The detection of the target protein was achieved by incubating the membrane with Clarity^™^ ECL Western Blotting Substrate (Bio-Rad Laboratories, Hercules, CA, USA) and analysed with ChemiDoc^™^ XRS system (Bio-Rad Laboratories, Hercules, CA, USA).

### RNA sequencing

The total RNA from the studied hTP cells: wild type (*n* = 3 experimental replicates); negative control (*n* = 3 experimental replicates) and *TKTL1* (*n* = 3 experimental replicates) was prepared for sequencing libraries according to the TruSeq Stranded Total RNA with Ribo-Zero (Illumina, San Diego, CA, USA) library protocol, and the high-throughput sequencing system, NovaSeq6000 (Illumina, San Diego, CA, USA) was used. To characterize the hTP cells, a comparative analysis of gene expression profiles was also conducted between non-modified hTP cells (*n* = 3) and commercial RNA from human testes with normal spermatogenesis (*n* = 3). The paired-end sequences were 2×150 bp in length. Expression data were deposited in the Gene Expression Omnibus, accession number GSE262557 and GSE262559. The sequenced reads were aligned to the GRCh38.p7 reference genome using HISAT2 2.0.5 (Kim et al., 2015), and FeatureCounts, which aligns reads within adequate GENCODE v25 gene annotation regions, were counted (Harrow et al., 2006; Liao et al., 2014). Finally, the read counts were normalized using DESeq2 in the R/Bioconductor environment (Love et al., 2014; Huber et al., 2015). The genes were selected according to the criteria described by Malcher et al. (2023), focusing on protein coded genes: at least a two-fold change (log2) in gene expression and with *P* < 0.05; expression level in the male gonad was verified using NCBI Gene, EMBL-EBI (Illumina Body Map), or both; expression level in gametogenic cells was checked using Human Protein Atlas; phenotypes, diseases and features associated with selected genes were checked using Ensembl; and the association with infertility, spermatogenesis, or both, was checked using *PubMed*.

### Immunofluorescence staining

#### For tissue samples

The formalin-fixed, paraffin-embedded tissue sections presenting normal spermatogenesis were incubated overnight with anti- TKTL1 rabbit polyclonal antibody (catalogue number ab155662) (Abcam, Cambridge, UK) 1:100; anti-UTF1 mouse monoclonal antibody (RRID:AB_2573041) (Invitrogen, Thermo Fisher Scientific, MT, USA) 1:50; anti-MLH1 mouse monoclonal antibody (RRID:AB_300987) (Abcam, Cambridge, UK) 1:30; anti-c-KIT monoclonal antibody (RRID:AB_11156422) (Thermo Fisher Scientific, MT, USA) 1:50; F-Actin (RRID:AB_302794) (Abcam, Cambridge, UK) 1:100 (diluted in 0.1% Triton-100 in 1x PBS) at 4°C. After rinsing 2 × 5 min with 1 × phosphate buffered saline (PBS), the sections were incubated with anti-rabbit Alexa Fluor 594 antibody (RRID:AB_2650602) and anti-mouse Alexa Fluor 488 antibody (RRID:AB_2576208), respectively (Abcam, Cambridge, UK) 1:400 (diluted in 1 × PBS) for 1 h at room temperature. After that, 20 *μ*l of DAPI was applied and the sample was covered with a coverslip. The protocol used was described by Malcher et al. (2023).

**FIGURE 2:**
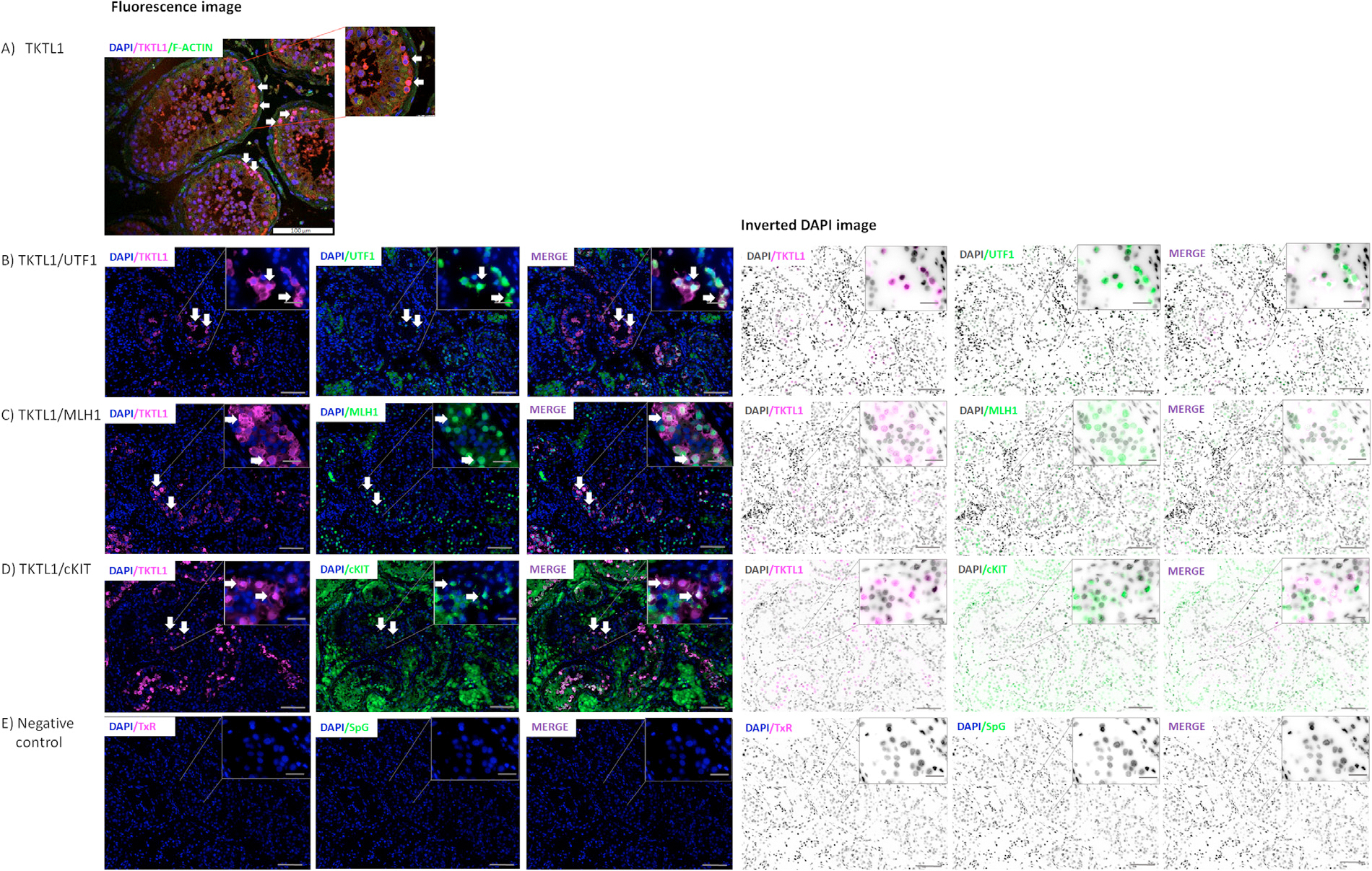
Localization of TKTL1 and specific spermatogonia (UTF1, MLH1, c-KIT) and cytoskeletal (F-Actin) markers in normal human testes tissue. Immunofluorescence and inverted DAPI images in human testes with normal spermatogonia production. Co-staining of TKTL1 (magenta) with (A) F-Actin (green); (B) UTF1 (green); (C) MLH1 (green); (D) c-KIT (green); (E) non-immune serum negative controls. Arrows indicate the TKTL1 positive staining. Counterstaining with DAPI. Images were acquired using (A) STELLARIS Leica confocal system; filters: DAPI/TxR/SpG/Triple; objectives 20× and 40×; software: LasXv4.6. (B–E) Leica DM5500 microscopy with a proper filter set (DAPI/TxR/SpG/Triple) was used; objectives: 10x, scale bar: 100 *μ*m, and 63x, scale bar: 20 *μ*m with oil immersion (insets); software: CytoVision. TxR - Texas Red; SpG - Spectrum Green.

For tissue samples, four different staining combinations were conducted with three technical replicates for each staining. The procedure was also carried out for negative controls, for which non-immune goat serum was used in place of the primary antibodies, and the remainder of the procedure was carried out as described above; the negative control results are presented in FIGURE 2.

Images were acquired using STELLARIS Leica confocal system; Leica DM5500; and Cytovision. STELLARIS Leica confocal system: filters: DAPI/TxR/SpG/Triple; objectives: 20× and 40×; software: LasXv4.6 as well as using a fluorescence microscope with a proper filter set; Leica DM5500: filters: DAPI/TxR/SpG/Triple; objectives: 10 × and 63 × with oil immersion; software: CytoVision. All images within an experiment were taken using the same settings.

For cell samples: The hTP cells, TCam-2 cells, or both, were fixed and stained according to the protocol described by Malcher et al. (2023). The cells were incubated overnight with anti-SOX17 ms (RRID:AB_1861437) (Abcam, Cambridge, UK) 1:50; anti-TFAP2C rb (RRID:AB_2891087) (Abcam, Cambridge, UK) 1:200; anti-DAZL rb (RRID:AB_2893177) (Abcam, Cambridge, UK) 1:200; anti-SPOCD1 rb (RRID:AB_11131573) (Abcam, Cambridge, UK) 1:200; anti-SAGE1 rb (RRID:AB_11139742) (Abcam, Cambridge, UK) 1:200; anti-SOX9 rb (RRID:AB_1080067) (Atlas Antibodies, Stockholm, Sweden)) 1:200; anti-CYP11A1 rb (RRID:AB_1310110) (Abcam, Cambridge, UK) 1:200; DDX4 (RRID:AB_443012) (Abcam, Cambridge, UK) 1:100 (diluted in 0.1% Triton-100 in 1 × PBS) at 4°C. After rising 2×5 min with 1 × PBS, the sections were incubated with anti-rabbit antibody (RRID:AB_2650602) (Abcam, Cambridge, UK) 1:500 or anti-mouse antibody (RRID: AB_2576208; Abcam, Cambridge, UK) 1:500 (diluted in 1 × PBS) for 1 h at room temperature. For cell samples, immunostaining was carried out in two experimental replicates for each sample studied. A Leica DMi8 with a proper filter set (DAPI/TxR/Double) was used; objectives: 40×; scale bar: 50 *μ*m; software: LASX. All images within an experiment were taken using the same settings.

### STRING gene interactions

The STRING database version 12.0 was used to indicate the possible interactions of the TKTL1 protein with the protein products of the selected genes from the RNA-sequencing analysis (Szklarczyk et al., 2023) (https://string-db.org/), including direct and indirect interactions between proteins, computational predictions, knowledge transfer between organisms and interactions aggregated from other databases. The interaction between *TKTL1* and the other genes in *Homo sapiens* was analysed knowing that STRING relies on well-annotated genomes from public repositories, e.g. Ensembl, UniProt, NCBI RefSeq. Published studies have focused on the interactions of proteins between TKTL1 and the selected proteins based on validated laboratory experiments (including co-immunoprecipitation, yeast two-hybrid assays), and their co-occurrence, as well as data from pathway and interaction databases, i.e. KEGG, BioGRID and Reactome.

### Statistical methods

Each experiment was conducted at least twice, and each experimental variant was reproduced in triplicate. For RNA-sequencing data, the differential analysis included computing of median-based fold change, statistical significance using the Student’s t-test, and false discovery rates (correction for multiple testing) in the R/Bioconductor programming environment (version 4.3.2; R Core Team R, 2024). GraphPad Prism7.03 statistical software was used to produce boxplot graphs. For real-time PCR, data were presented as mean with SD, and analysed using one-way analysis of variance, Tukey’s multiple comparisons test and presented as column graphs by GraphPad Prism7.03 statistical software.

## RESULTS

### Localization of the TKTL1 gene product in human testes

The immunofluorescence analysis showed that the TKTL1 protein is located in the nuclei of spermatogonia and preleptotene spermatocytes in normal human testicular tissue with maintained spermatogenesis (FIGURE 2A). The co-staining of TKTL1 with UTF1, a marker of spermatogonial stem cells (undifferentiated spermatogonia in in state 0 and 1), revealed the localization of the TKTL1 protein in the nuclei of undifferentiated spermatogonia. Not all UTF1-positive cells, however, showed TKTL1 expression (FIGURE 2B). The co-staining of TKTL1 with MLH1 (in state 1–4 spermatogonia as well as preleptotene spermatocytes) showed co-localization of these proteins in the nuclei, and in the cytoplasm of spermatogonia and preleptotene spermatocytes (FIGURE 2C). Some individual cells presented a strong signal for either TKTL1 or MLH1 protein (FIGURE 2C). Staining for TKTL1 together with c-KIT protein, used as a marker for differentiated spermatogonia, indicated a cytoplasmatic localization for c-KIT and a nuclear presence for TKTL1. Some individual cells were positive only for TKTL1 protein or with stronger signal for c-KIT (FIGURE 2D). The c-KIT also showed a strong expression in Leydig cells, which was not observed for TKTL1 (FIGURE 2D).

### Characterization of the human testicular primary cells

Gene expression profile analysis was conducted to compare hTP cells and adult testes with normal spermatogenesis using RNA-sequencing to determine the specific testicular cell markers that may characterize this cell population. The heatmap showed that hTP cells express genes characteristic of somatic cells and spermatogonia, and most of the presented genes were expressed in undifferentiated spermatogonia (Supplementary Figure 1A). Their expression in the examined cells was similar to those from normal testes (Supplementary Figure 1A). The expression of some genes demonstrating a profile from later stages of spermatogenesis characteristic of spermatocytes, spermatids and spermatozoa were not expressed in the human testicular primary cells (Supplementary Figure 1A). Using real-time PCR analysis, hTP cells were compared with adult testes with normal spermatogenesis and with TCam2 cells (a testicular seminoma cell line), with expression of a number of early and late germ cells markers; however, their disadvantage was the abnormal karyotype, including some chromosomal gain or deletion, or loss of restricted regions. The hTP and TCam-2 cells do not express the *TKTL1* gene, which is present in the human testes with normal spermatogenesis (*P* = 0.0147) (Supplementary Figure 1B). The hTP cells had features of undifferentiated spermatogonia, and the expression level of the *SPOCD1* gene, specific for undifferentiated spermatogonia, was comparable to that observed in male gonadal tissue with normal spermatogenesis; this was not the case with TCam2 cells, which has no expression of this gene (*P* ≤ 0.0146) (Supplementary Figure 1B). The *CD68* and *MKI67* genes, characterizing spermatogonia, demonstrated similar expression levels in hTP cells and normal male gonadal tissue, whereas TCam-2 cells manifested a weaker expression of the *CD68* gene (*P* ≤ 0.0231), and a two-fold decrease in the *MKI67* gene expression level compared with hTP cells (Supplementary Figure 1B). These results were confirmed by immunofluorescence staining (Supplementary Figure 1C). Strong staining was presented for SPOCD1 protein in hTP cells, whereas, in the case of DAZL, SAGE1, DDX4 proteins, which are characteristic of differentiated spermatogonia, a positive signal was observed only in sporadic cells of hTP cells (Supplementary Figure 1C). The hTP cells also showed a positive signal for TFAP2C and SOX17 (markers of primordial germ cells, driving the human germ-cell specification programme) and SOX9 (Sertoli cells marker) proteins.

### Activation of the *TKTL1* gene by transfection of TCam-2 cells with the CRISPR system

Following the antibiotic selection and the induction with doxycycline (after 72 h), transfection efficiency was estimated for both TCam-2 with the activated *TKTL1* gene and for TCam-2 with non-specific gRNAs (guide RNAs), whereas, for the human genome, the negative control TCam-2, was of 37% and 43%, respectively (Supplementary Figure 2A). After 72 h, upregulation was observed (*P* ≤ 0.004) of the *TKTL1* gene at the mRNA level in *TKTL1*-activated TCam-2 samples (marked as TKTL1) compared with both control groups: TCam-2 wild type and the negative control (Supplementary Figure 2B). The expression level of the respective protein, however, remained low, and weak bands were observed in the Western blot analysis of the *TKTL1*-activated TCam2 cells (marked as TKTL1) (Supplementary Figure 2C); therefore, the experiments did not proceed further using this cell line.

### Overexpression of the *TKTL1* gene in human testicular primary cell suspension

**FIGURE 3:**
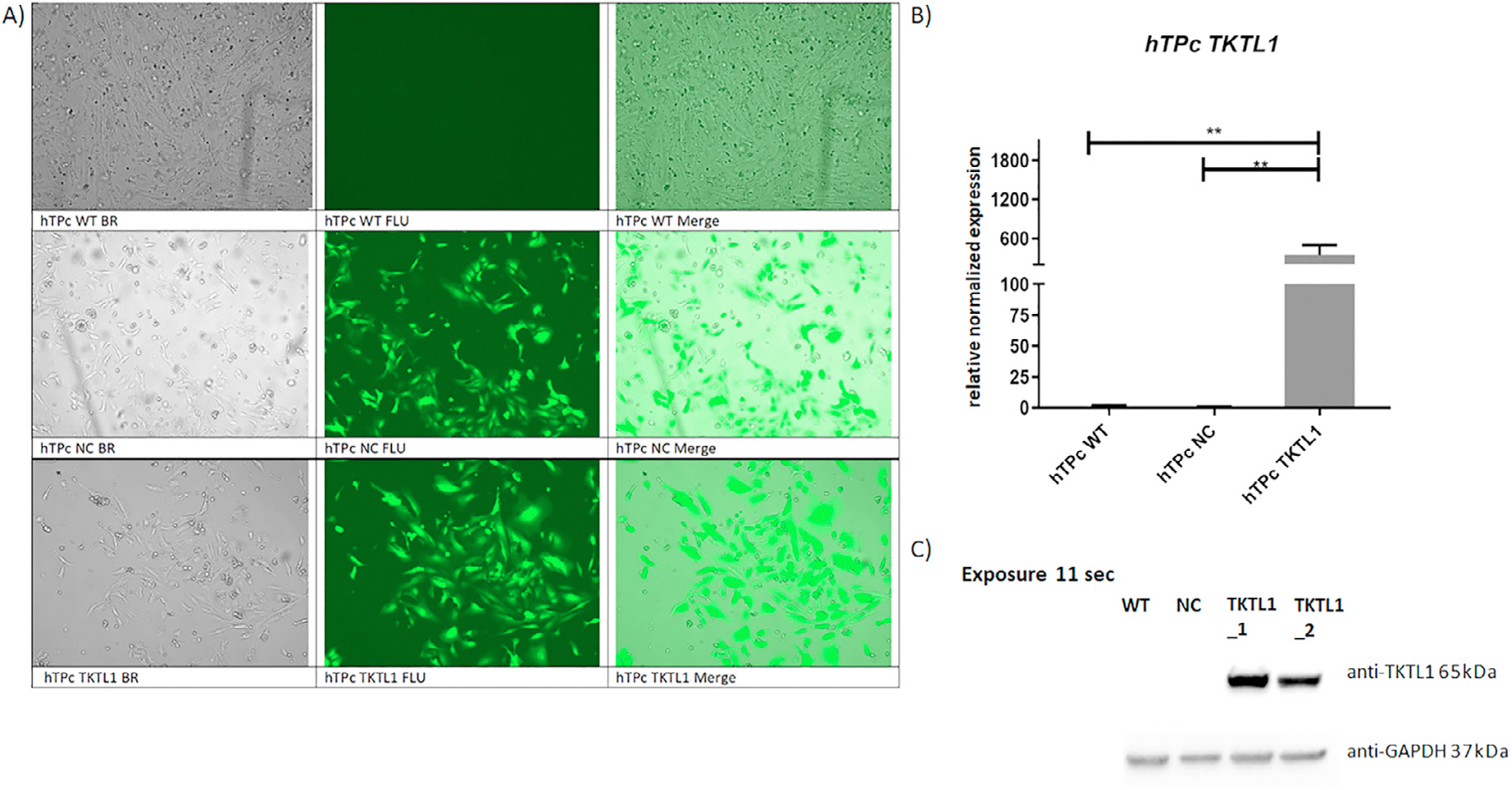
Overexpression of the *TKTL1* gene using the lentiviral system in human testicular primary (hTP) cells. (A) The hTP cells at 72 h after transduction in a Juli FL fluorescence microscope; scale bar: 100 *μ*m; (B) the real-time polymerase chain reaction analysis of *TKTL1* gene expression. Data presented as mean + SD. *P*-values are presented on each graph. Data analysed by one-way analysis of variance with Tukey’s multiple comparisons test; (C) representative Western blot analysis of TKTL1 protein expression, exposure time 11 s, and its graph of the relative quantity of TKTL1 protein normalized with reference to GAPDH analysed by Image Lab 6.1 tools. BR brightfield, FLU fluorescence; NC, negative control with GFP expression; TKTL1, cells with overexpression of *TKTL1* gene; WT, wild type cells in in-vitro culture medium only.

The transduction efficiency was estimated after 72 h. About 75% of GFP-positive cells was observed for both studied groups: hTP cells with overexpression of TKTL1 gene as well as negative control with only GFP sequence (FIGURE 3A). *TKTL1* overexpression was verified after the antibiotic selection (after 1 week) at the mRNA level using real-time PCR. An impressive increase was observed in the expression of *TKTL1* gene in the modified hTP cells with *TKTL1* overexpression compared with wild type and negative control, which do not express *TKTL1*normally (*P* = 0.008) (FIGURE 3B). The result was also confirmed at respective protein levels by Western blotting, and a strong band of TKTL1 protein was observed in the modified hTP cells with *TKTL1* overexpression, whereas no band was visible in both controls applied (FIGURE 3C).

### Pool of genes differently expressed by *TKTL1* gene overexpression in human testicular primary cells

**FIGURE 4:**
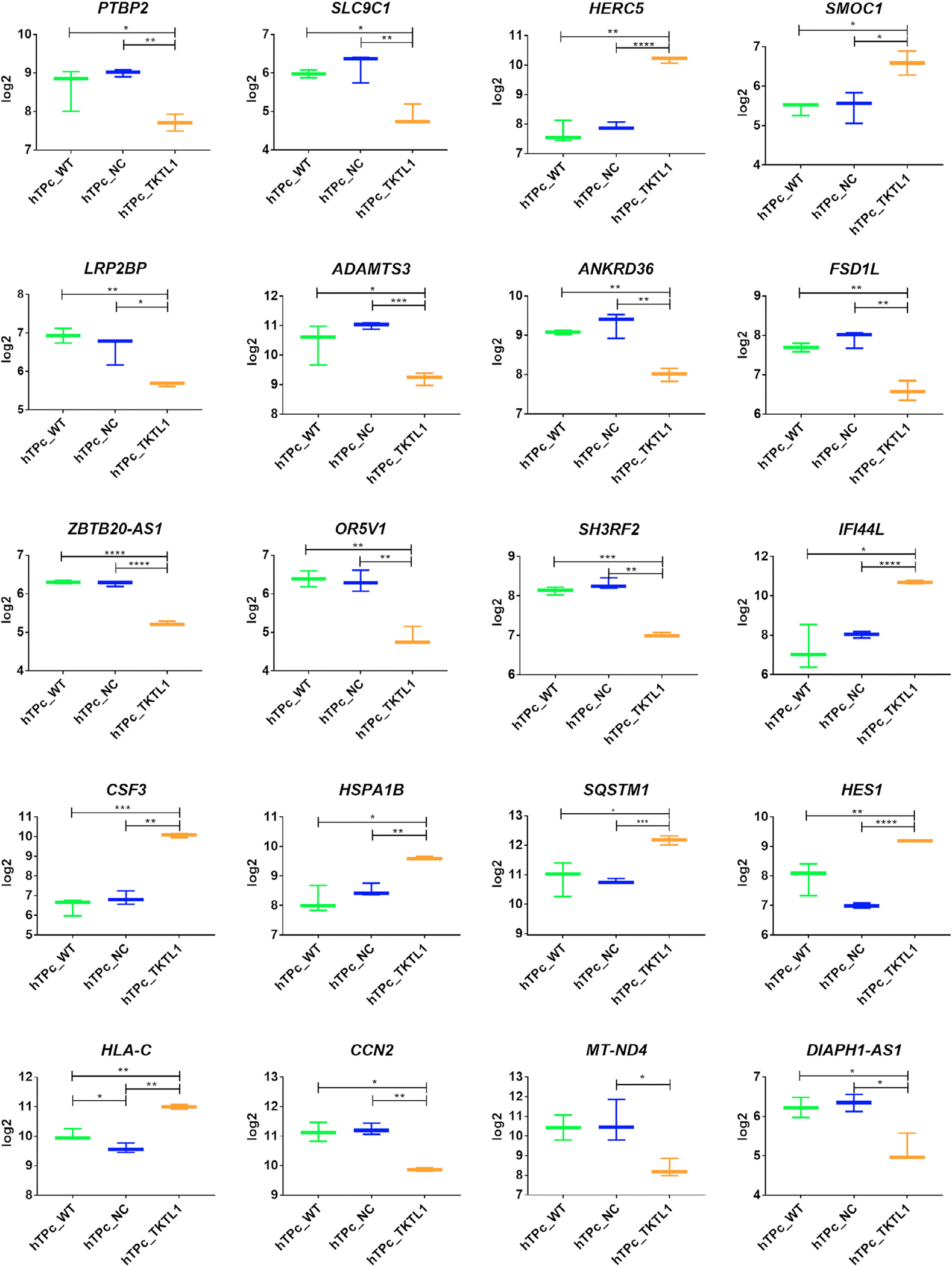
The 20 selected genes assessed in human testicular primary (hTP) cells with overexpressed *TKTL1* gene and controls analysed by RNA-sequencing with a minimum of a twofold change and *P* < 0.05 in hTP cell suspension with overexpression of *TKTL1* compared with both applied controls. NC, negative control with GFP (green fluorescent protein) expression (*n* = 3 experimental replicates); TKTL1, cells with the overexpressed *TKTL1* gene (*n* = 3 experimental replicates); TKTL1, cells with the overexpressed *TKTL1* gene (*n* = 3 experimental replicates); WT, wild type cells in in-vitro culture medium only (*n* = 3 experimental replicates). The box plots represent the distribution of log2 expression with the median indicated by the centre line, and the interquartile range representing the middle 50% of values. *P*-values are presented on each graph. Data analysed by the Student’s t-test, and false discovery rates (correction for multiple testing).

First, the overexpression of the *TKTL1* gene was confirmed in human testicular primary cells by RNA sequencing (Supplementary Figure 3A). A total of 63,857 sequence tags was obtained by RNA sequencing and 204 differently expressed genes were identified after the criteria of at least two-fold change and *P* < 0.05. Then, out of these 204 differentially expressed genes, the top 20 genes associated with spermatogenesis were selected. In these genes, expression most significantly differed in the genetically modified hTP cells with overexpression of *TKTL1* gene compared with both controls to determine the relationship with spermatogenesis (Supplementary Figure 3B and Supplementary Figure 3C). The genes: *HERC5* (HECT and RLD domain containing E3 ubiquitin protein ligase 5), *SMOC1* (SPARC related modular calcium binding 1), *IFI44L* (interferon induced protein 44 like), *CSF3* (colony stimulating factor 3), *HSPA1B* (heat shock protein family A [Hsp70] member 1B), *SQSTM1* (sequestosome 1), *HES1* (hes family bHLH transcription factor 1) and *HLA-C* (major histocompatibility complex, class I, C) were upregulated with a minimum of a twofold change (all *P* < 0.05) in cells with overexpression of *TKTL1*, whereas such genes as: *PTBP2* (polypyrimidine tract binding protein 2), *SLC9C1* (solute carrier family 9 member C1), *LRP2BP* (LRP2 binding protein), *ADAMTS3* (ADAM metallopeptidase with thrombospondin type 1 motif 3), *DIAPH1-AS1* (DIAPH1 antisense RNA 1), *ANKRD36* (ankyrin repeat domain 36), *FSD1L* (fibronectin type III and SPRY domain containing 1 like), *ZBTB20-AS1* (ZBTB20 antisense RNA 1), *OR5V1* (olfactory receptor family 5 subfamily V member 1), *SH3RF2* (SH3 domain containing ring finger 2), *CCN2* (cellular communication network factor 2) and *MT-ND4* (mitochondrially encoded NADH dehydrogenase 4) were downregulated (all *P* ≤ 0.05) in the *TKTL1*-overexpressed cells compared with controls (FIGURE 4). Despite the lack of a known and clear interaction between TKTL1 and SQSTM1, HSPA1B, CCN2, ADAMTS3, HERC5 and IFI44L genes in the STRING database, overexpression of *TKTL1* induced change in the expression level of the named genes. The SQSTM1, HSPA1B, CCN2, ADAMTS3 interact together, according to STRING database, whereas HERC5 interacted only with IFI44L (Supplementary Figure 3D); these genes have been involved in the cell cycle regulation through two different pathways.

### Verification of the gene expression level in human testicular primary cells by real-time polymerase chain reaction

**FIGURE 5:**
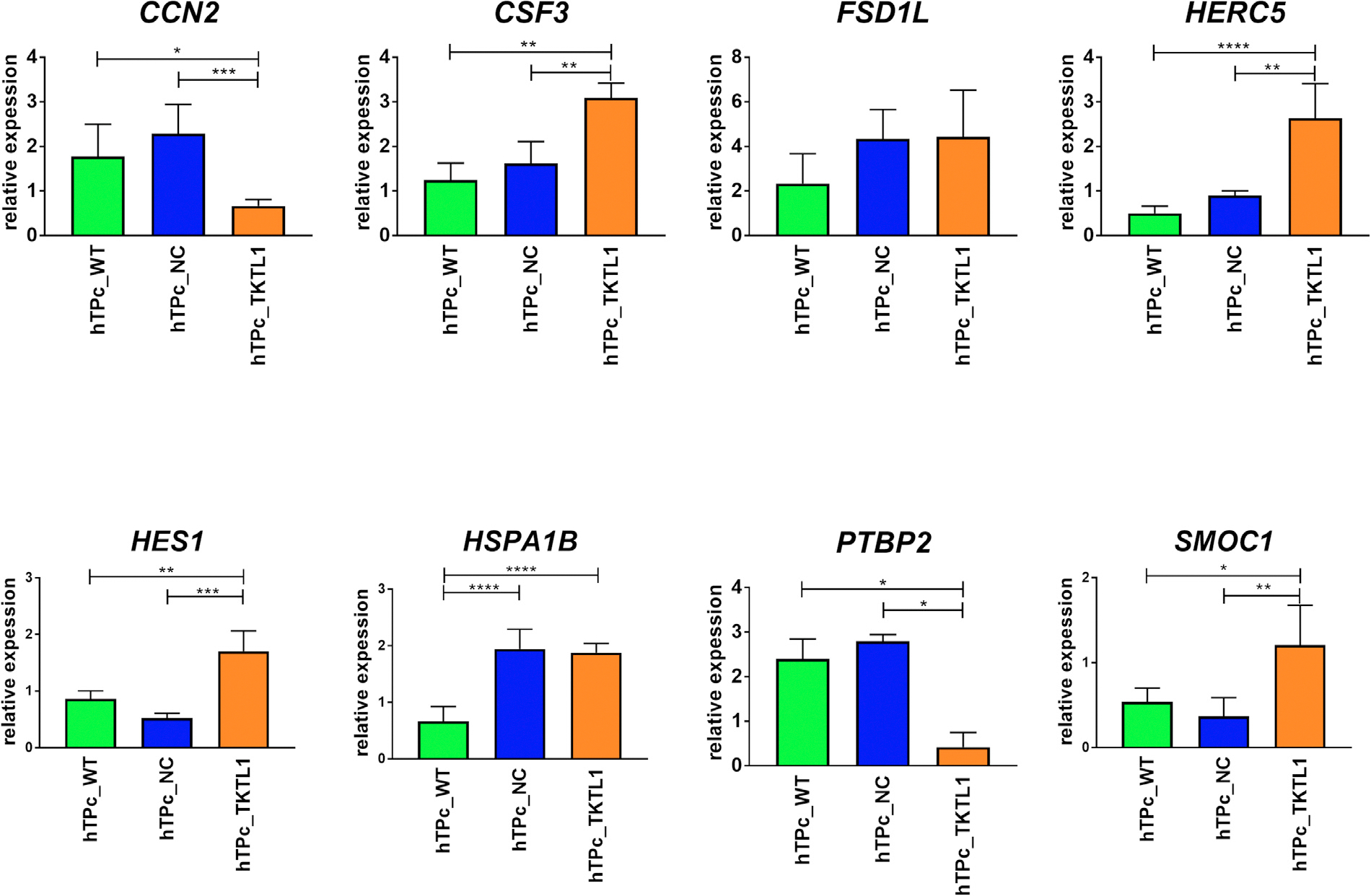
Verification of selected genes by real-time polymerase chain reaction (*CCN2, CSF3, FSD1L, HERC5, HES1, HSPA1B, PTBP2, SMOC1*) in human testicular primary (hTP) cells with overexpressed *TKTL1* gene versus controls in hTP cells. NC, negative control with GFP (green fluorescent protein) expression (*n* = 3 experimental replicates); TKTL1, cells with the overexpressed *TKTL1* gene (*n* = 3 experimental replicates); WT, wild type cells in in-vitro culture medium only (*n* = 3 experimental replicates), Data presented as mean + SD. *P*-values are presented on each graph. Data analysed by one-way analysis of variance with Tukey’s multiple comparisons test.

The list of genes analysed in the RNA-sequencing data were narrowed to eight selected differently expressed genes (*CCN2, CSF3, FSD1L, HERC5, HES1, HSPA1B, PTBP2, SMOC1*), which are involved in the regulation of the cell cycle, from the proliferation to apoptosis. Because their function is similar to the role of *TKTL1* in different cancer types, it was hypothesized that these genes could be directly or indirectly regulated by *TKTL1* during spermatogenesis. The gene expression analysis in hTP cells using real-time PCR confirmed the results obtained in the RNA-sequencing analysis for six of these genes. The overexpression of the *TKTL1* gene in hTP cells led to an increased expression of the following genes: *HERC5* (*P* ≤ 0.01), *SMOC1* (*P* ≤ 0.0056), *CSF3* (*P* ≤ 0.0042) and *HES1* (*P* ≤ 0.0034) compared with both controls (FIGURE 5). On the other hand, for both *PTBP2* and *CCN2* genes, their expression level was downregulated (*P* ≤ 0.0197) compared with the controls (FIGURE 5). For the *HSPA1B* gene, its expression level increased significantly in the cells with *TKTL1* overexpression compared with wild type cells (*P* < 0.0001). This was not confirmed, however, when the negative control was compared with GFP. The real-time PCR for the *FSD1L* gene did not confirm its expression level obtained from RNA-sequencing analysis (FIGURE 5).

### Verification of gene expression levels in patients with non-obstructive azoospermia

**FIGURE 6:**
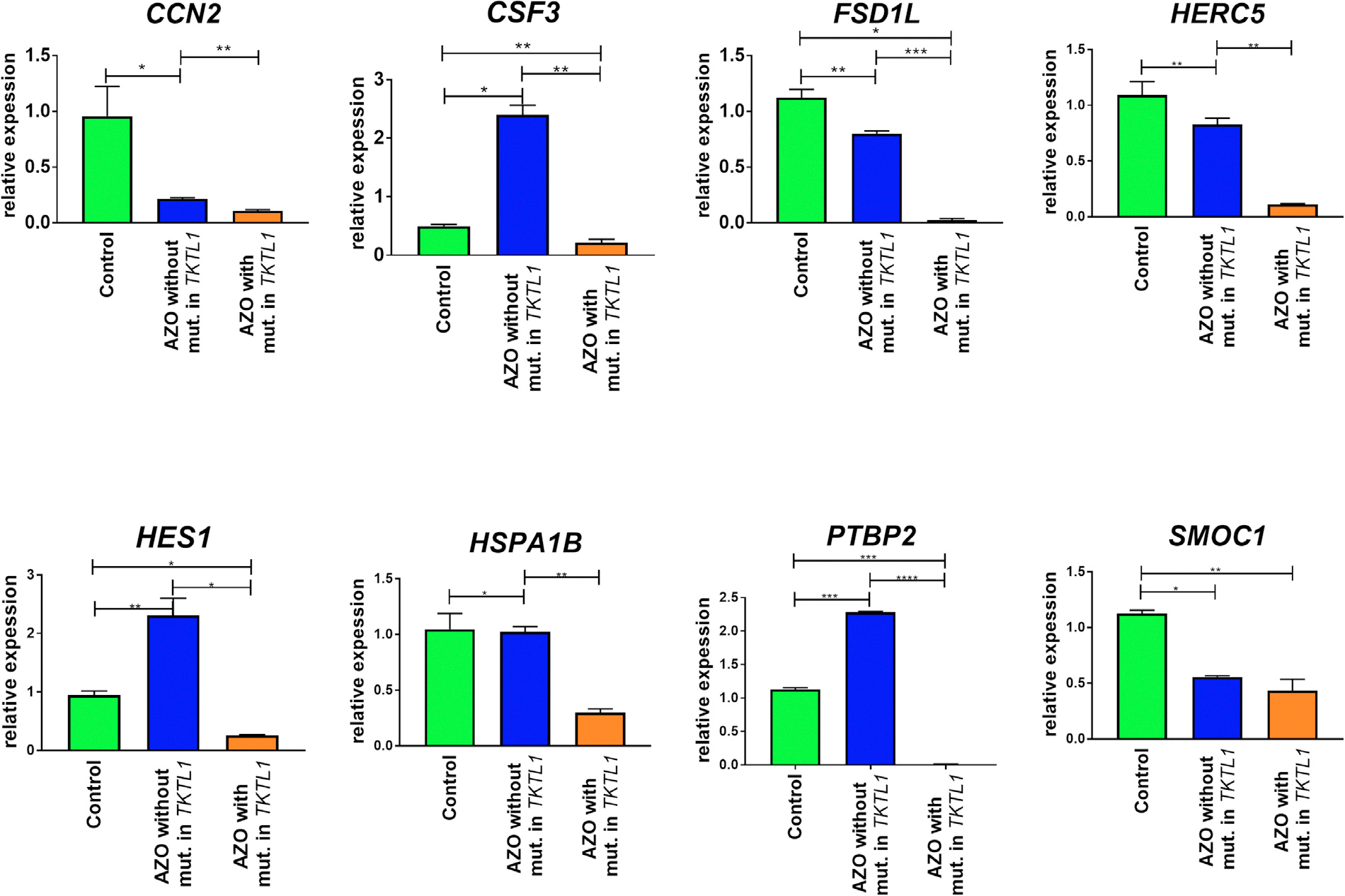
Verification of selected genes by real-time polymerase chain reaction (*CCN2, CSF3, FSD1L, HERC5, HES1, HSPA1B, PTBP2, SMOC1*) in testicular tissue from patients with non-obstructive azoospermia (NOA) and controls. Control, male gonadal tissue from men with normal spermatogenesis (*n* = 2 technical repeats); NOA *TKTL1*, patient with NOA without mutation in the *TKTL1* gene (*n* = 2 technical repeats); NOA *TKTL1*^mut^, patient with NOA with mutation in the *TKTL1* gene (*n* = 2 technical repeats). Data presented as mean + SD. *P*-values are presented on each graph. Data analysed by one-way analysis of variance with Tukey’s multiple comparisons test.

The eight selected differentially expressed genes (*CCN2, CSF3, FSD1L, HERC5, HES1, HSPA1B, PTBP2, SMOC1*) were also verified in patients with NOA. The real-time PCR was carried out with male gonad tissue samples from an azoospermic patient carrying a mutation in the *TKTL1* gene compared with an azoospermic patient without mutation in the *TKTL1* gene (negative control), and with control samples with normal spermatogenesis. The results showed a significant downregulation of the *CSF3, FSD1L, HERC5, HES1, HSPA1B, PTBP2* genes (all *P* ≤ 0.05) (FIGURE 6). The *SMOC1* and *CCN2* gene expression showed significant difference between patients with NOA with *TKTL1* mutation and controls with normal spermatogenesis but not compared with patients with NOA without *TKTL1* mutation.

## DISCUSSION

The aim of the present study was to investigate the function of *TKTL1* in spermatogenesis. *TKTL1* may cause NOA indirectly through its potential role in regulating the proliferation of germ cells and protecting their growth and survival.

### Potential causative variants in TKTL1 gene in patients with non-obstructive azoospermia and the role of TKTL1 in carcinogenesis

In the past decade, owing to highthroughput techniques and genome editing methods, many genetic causes of NOA were discovered. Among these is the *TKTL1* gene, for which we have previously identified two ultra-rare pathogenic variants: c.268_268delG and c.1601A>G using whole genome sequencing, in two patients: one with premeiotic and the second with meiotic arrest (Malcher et al., 2022). According to the structural protein modelling for the TKTL1 that was previously conducted, the first variant, c.268_268delG (p.Asp90Met fs*35), resulted in the mutation of a negatively charged aspartic acid residue to a hydrophobic amino acid, and truncated most of the protein (Malcher et al., 2022). The second variant, c.1601A>G (p. Glu534Gly), led to amino acid change in the C-terminal domain, suggesting that this mutation destabilizes the folding in this domain because the glutamic acid at this position has been predicted to stabilize this domain (Malcher et al., 2022).

Following the publication of our previous results (Malcher et al., 2022), Khan et al. (2023) conducted a study of seven large families from Pakistan with clinically diagnosed male infertility. An in-frame hemizygous deletion was detected in the *TKTL1* gene of brothers with spermatogenic impairment (referred to GRCh38: chrX:154305324_154305327del). Moreover, five azoospermic cases from GEMINI cohort (Nagirnaja et al., 2022; Riera-Escamilla et al., 2022) were reported to have other deleterious variants in the *TKTL1* gene (Khan et al., 2023). Those independent findings of mutations in the *TKTL1* gene and the degree of spermatogenesis impairment in patients (mainly with NOA) carrying these mutations may indicate an important role for *TKTL1* in the process of spermatogenesis. Apart from the mentioned studies, it was also demonstrated that TKTL1 protein may be a biomarker distinguishing semen between fertile men and men with NOA (Rolland et al., 2013). In addition, the overexpression of *TKTL1* in several human cancers may lead to the transformation of substrates necessary for glucose degradation (in the glycolysis pathway) (Zhang et al., 2007a; da Costa et al., 2018; Li et al., 2019).

### TKTL1 protein localization in normal testicular tissue

It is essential to note that *TKTL1* gene is highly and exclusively expressed in the testes (median TPM 167.9 [data from *GTEx* database]), and our immunofluorescence staining of TKTL1 with specific spermatogonia markers (UTF1, MLH1 and c-KIT) showed the positive signal predominantly in the nuclei of spermatogonia from state 1 to 4 as well as in preleptotene spermatocytes (FIGURE 2). The co-staining of TKTL1 with UTF1, a specific undifferentiated spermatogonia marker (in state 0 and 1), showed a positive stain of the TKTL1 protein spermatogonia in state 1 but not 0, suggesting that TKTL1 protein expression starts at the state 1 of the undifferentiated spermatogonia. The TKTL1 protein expression remained positive until spermatogonia state 4, verified by the co-staining of TKTL1 with c-KIT, a marker of spermatogonia in state 2 and 3, and preleptotene spermatocytes, as these were observed in the co-staining of TKTL1 with MLH1. Our results confirmed the data obtained from single cell RNA-sequencing analysis in the *Human Protein Atlas database*, showing that TKTL1 is expressed specifically at the stage of spermatogonia and preleptotene spermatocytes.

### Studies of the TKTL1 gene in in-vitro models

To shed more light on the function of *TKTL1* gene in human spermatogenesis, its overexpression was induced in hTP cells, which do not express the *TKTL1* gene, and the obtained results were verified using the male gonad tissue samples of patients with NOA to uncover the genes cluster regulated by *TKTL1*. An attempt was also made to activate the *TKTL1* gene in TCam-2 cells using the CRISPR system; however, despite the significant activation of this gene at mRNA level, a satisfactory result of *TKTL1* expression at the protein level was not obtained. Therefore, further research was focused on the overexpression of *TKTL1* in hTP cells, in which its expression was significantly increased at mRNA and protein levels. It is acknowledged that both these cell types are not the ideal model to study spermatogenesis, especially because hTP cells with further proliferation will show characteristics that are closer to somatic cells than the germ cells one. These cells, however, are one of the best options currently available on the market for this purpose. In addition, none of the available cell types to study spermatogenesis express *TKTL1*, rendering our technique to overexpress *TKTL1* gene in hTP cells and determining its effect on the network of genes expressed in testes a good one to partially understand the *TKTL1* gene function.

### Pool of genes potentially regulated by TKTL1

The expression level analysis of the *TKTL1* gene in hTP cells by RNA sequencing showed an over 60-fold increase in hTP cells with overexpressed *TKTL1* gene compared with control samples applied (*P* < 0.0001) (FIGURE 3). The comparative gene expression profile analysis between hTP cells with overexpressed *TKTL1* gene and control cells, following the criteria of at least two-fold change and *P* < 0.05, allowed the identification of 204 differently expressed genes distinguished from all 63,857 sequence tags obtained by RNA sequencing. Selecting the genes expressed in the testes and germ cells, the pool was reduced to 20 genes (FIGURE 4 and Supplementary Figure 3). On the basis of the role of *TKTL1* gene, elucidated in previous cancer studies, encoding an enzyme involved in the non-oxidative pentose-phosphate pathway (which plays a crucial role in supporting cell proliferation by contributing to nucleotide synthesis through the production of ribose-5-phosphate (Diaz-Moralli et al., 2016), the focus narrowed to eight crucial genes: *HERC5, CSF3, HES1, HSPA1B, CCN2* (Nakanishi et al., 2001; Chen et al., 2020; Nishida and Kubota, 2020), *FSD1L* (Gao et al., 2022; Taschereau et al., 2023), *PTBP2* (Zagore et al., 2015; Diaz-Moralli et al., 2016; Hannigan et al., 2017; Pina et al., 2018; Liu et al., 2021), *SMOC1* (Gao et al., 2019; Montgomery et al., 2020), involved in the regulation of cell cycle, from proliferation to apoptosis. This suggests that *TKTL1* may be essential for cell cycle regulation by modulating the expression of these genes, either directly or indirectly, and, therefore, supporting our hypothesis.

### Verification of the potentially regulated genes in modified human testicular primary cells and in patients with nonobstructive azoospermia with and without mutation in TKTL1

In conducting the expression analysis of these genes in the male gonad tissue, it was expected that the sample collected from a patient with NOA without a mutation in the *TKTL1* would present similar expression to the control samples (the samples obtained from men with normal spermatogenesis), whereas, the samples from a patient with NOA with a mutation in the *TKTL1* gene would show an inverse relationship with the expression level of the studied genes compared with the genetically modified hTP cells with an activated *TKTL1* gene. The expected result was obtained for the *HERC5, CSF3, HES1* and *HSPA1B* genes (FIGURE 5 and FIGURE 6), in which a minimum 3.5-fold downregulation (*P* < 0.05) was observed in samples of patients with a mutation in the *TKTL1* gene compared with both control samples in accordance with the results obtained for hTP cells.

### Potential function of TKTL1 in human spermatogenesis

We could hypothesize that the *TKTL1* gene plays a role in proliferation at spermatogonial stage and protects the growth and survival of germ cells by attenuating reactive oxygen species (ROS) secretion, via *HERC5* and *CSF3* genes. In fact, the *HERC5* gene, despite not being defined as spermatogenesis related gene, is highly expressed in the testes compared with the other tissues, and it mediates the proliferation and migration of malignant cells, in non-small cell lung cancer and breast cancer by interacting with the p53 protein (Liu et al., 2022). The *Human Protein Atlas* data showed that *HERC5* expression level is highest in spermatogonia. Hence, the *TKTL1* gene together with the *HERC5* may be responsible for the spermatogonial stages proliferation (Schultz et al., 2008). On the other hand, the *CSF3* gene belongs to the cytokines group that regulates the germ cells proliferation in male reproductive system and differentiation of mesenchymal cells (Syriou et al., 2018). The *CSF3* gene expression has also been correlated with semen quality parameters, such as sperm concentration, motility, viability and morphology (Jiang et al., 2016). It has been observed that the expression of this gene is increased in semen samples with high levels of ROS release (Jiang et al., 2016). The pentose phosphate pathway, in which the *TKTL1* gene is most likely involved, produces NADPH, which is a key element in carrying out the reduction reaction avoiding the cell degradation by ROS (Diaz-Moralli et al., 2016). Therefore, it is suggested that the *CSF3* gene may act together with *TKTL1* reducing ROS production, harmful to cell growth and survival (Schieber and Chandel, 2014).

*TKTL1* may be also involved in regulating the balance between germ cells apoptosis and proliferation, as it was found that *TKTL1* affects the expression level of both *HES1* and *HSPS1B*. In fact, the *HES1* gene plays a key role in many physiological processes, including cell differentiation, cell cycle arrest, apoptosis, the ability to self-regeneration, or all (Liu et al., 2015). In mice, the expression of the *Hes1* gene is regulated by the Notch signalling pathway, and activity in this pathway is required for normal spermatogenesis (Hasegawa et al., 2012). Blockade of Notch signalling disrupts the expression of its components, including the *HES1* gene, and causes disturbances in the proliferation and the function of male germ cells, significantly increasing apoptosis, mainly in the last stages of spermatogenesis. This may contribute to the formation of morphological defects in spermatozoa (Murta et al., 2014). It was shown that overexpression of the *TKTL1* gene in the process of carcinogenesis protects cancer cells against apoptosis, and the elimination of *TKTL1* leads to inhibition of proliferation of malignant cells (Zhang et al., 2007b; Diaz-Moralli et al., 2016). Therefore, it is suggested that the *HES1* gene together with the *TKTL1* gene may be responsible for regulating the balance between the proliferation and apoptosis of germ cells. Finally, the *HSPA1B* gene has been involved in reducing the harmful effect of hyperthermia on spermatogenesis and post-ejaculatory sperm function (Ji et al., 2014). The *HSPA1B* can protect germ cells against various stresses by inhibiting stress-induced apoptosis (Ji et al., 2014), which is similar to the function of *TKTL1* gene as a cytoprotective agent against apoptosis in carcinogenesis (Diaz-Moralli et al., 2016).

### Limitations of the study

The present study has some limitations, most notably the small number of NOA samples containing *TKTL1* mutation. Moreover, the main drawback of the hTP cells was that they did not express the *TKTL1* gene and, therefore, it was impossible to conduct a knock-out experiment. Therefore, we conducted an experiment to induce the overexpression of *TKTL1* in these cells and indirectly investigate its function. The hTP cells are currently one of the only available cells that can be used to study gene functions in the aspect of human spermatogenesis, as no appropriate model is currently available to study gene functions in human spermatogenesis, among others *TKTL1*. Equally, it was not possible to study the function of *TKTL1* using mouse models, despite human TKTL1 and mouse Tktl1 protein sharing significant sequence homology, because of notable differences in specific regions, especially in functional domains that may be critical for the function of the TKTL1 protein (Schneider et al., 2012; Pinson et al., 2022). It was even observed that Tktl1-null knockout mice had no reproductive defects or effect on fertilization (Miyata et al., 2016), whereas ours, as well as other research groups, observed that patients with *TKTL1* mutation presented with NOA phenotype (Malcher et al., 2022; Nagirnaja et al., 2022; Riera-Escamilla et al., 2022; Khan et al., 2023).

In conclusion, mutations in genes responsible for the process of spermatogenesis may decrease the proliferation of germ cells and disturb their course. The conducted research in the NOA patient sample with a mutation in the *TKTL1* gene showed a decreased expression level in genes involved in the regulation of cell proliferation and apoptosis (*CSF3, HERC5, HES1, HSPA1B*), which suggest that the *TKTL1* may be involved in the regulation of gametogenesis affecting the proliferation and differentiation of germ cells at the early stages. Although at this stage it is difficult to conclude whether *TKTL1* is essential for a successful spermatogenesis, this study may provide a further understanding of genomic cases of male infertility contributing to the platform of genes that should be recognized as causative of NOA. An international effort to create such a platform for future diagnosis and treatment of azoospermia is under way.

Functional studies are still required to test our hypothesis, and, because of the limitations mentioned previously, we plan to conduct thorough studies to better understand the mechanisms of TKTL1 using spatial transcriptomic analysis on testicular tissues from patients bearing *TKTL1* mutations, which would provide information about the expression profile of this gene, and insight into its function in spermatogenesis. We will also generate an in-vitro model based on induced pluripotent stem cells from patients with NOA-bearing causative variants in *TKTL1*, which will be corrected using CRISPR technology to study the effect of this correction on cell differentiation.

## Supplementary Material

MMC1

Supplementary material associated with this article can be found in the online version at doi:10.1016/j.rbmo.2025.104895.

## KEY MESSAGE

The mutation of *TKTL1* gene showed a decreased expression level of genes involved in the regulation of cell proliferation and apoptosis (*CSF3, HERC5, HES1, HSPA1B*), which suggests that the *TKTL1* may be also involved in the regulation of gametogenesis affecting the proliferation and differentiation of germ cells at the early stages.
