# Erratum to TKTL1: a new candidate gene in non-obstructive azoospermia Reproductive BioMedicine Online 51/3 (2025) 104895

**DOI:** 10.1016/j.rbmo.2025.105185

**Published:** 2025-08-09

**Authors:** Agnieszka Malcher, Mikołaj Smolibowski, Tomasz Stokowy, Hermann Bauer, Alicja Patyk, Piotr Jedrzejczak, Jagoda Kostyk, Zuzanna Graczyk, Rim Ibrahim, Katarzyna Bednarek-Rajewska, Anna Berger, Alexander N. Yatsenko, Maciej Kurpisz

**Affiliations:** a Institute of Human Genetics, Polish Academy of Sciences, Poznan, Poland; b IT Division, University of Bergen, Bergen, Norway; c Department of Developmental Genetics, Max Planck Institute for Molecular Genetics, Berlin, Germany; d Center of Obstetrics, Gynecology and Infertility Treatment – Pastelova Clinic, Poznan, Poland; e Department of Cell Biology, Poznan University of Medical Sciences, Poznan, Poland; f Department of Clinical Pathology, Poznan University of Medical Sciences, Poznan, Poland; g Department of Obstetrics and Gynecology and Reproductive Sciences, School of Medicine, University of Pittsburgh, Pittsburgh, PA, USA

The publisher regrets that the revised figures were not correctly included in the published article. The correct, revised FIGURES 2, 3, 4, 5, and 6 are shown below with the associated captions. The publisher would like to apologise for any inconvenience caused.

## Figures and Tables

**FIGURE 2:**
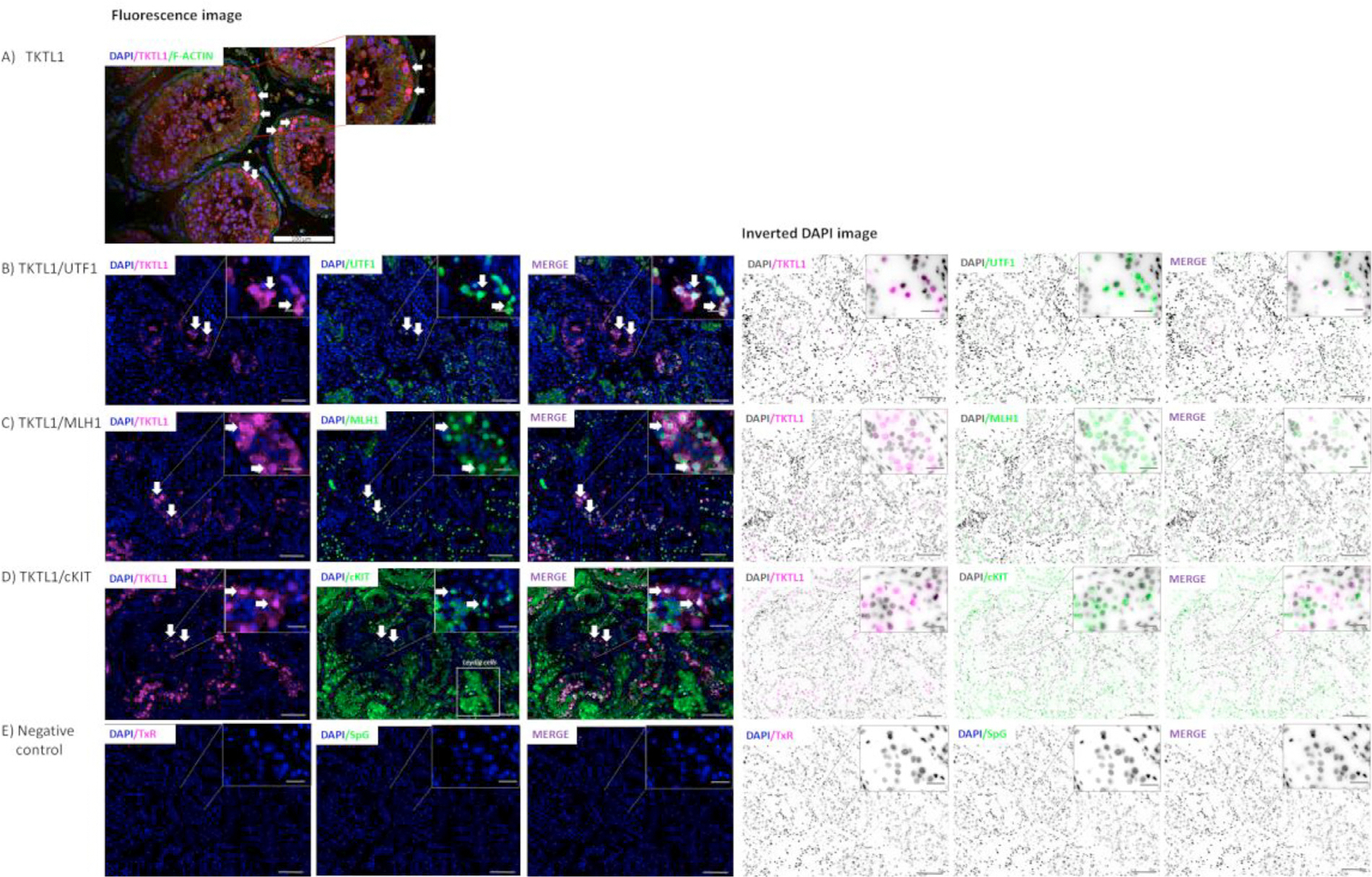
Immunofluorescence and inverted DAPI image of TKTL1 localization in normal human testis. A) Co-staining of TKTL1 with F-actin in normal testis tissue analysed using Images were acquired using a STELLARIS Leica confocal system, filters: DAPI/TxR/SpG/Triple; objectives 20 × and 40 ×; software: LasXv4.6. Arrows indicate the TKTL1 positive staining. B) Co-staining of TKTL1 with UTF1. C) Co-staining of TKTL1 with MLH1. D) Co-staining of TKTL1 with c-KIT. E) Negative Response to Reviewers 2 control. Leica DM5500 microscopy with a proper filter set (DAPI/TxR/SpG/Triple) was used; objectives: 10x, scale bar: 100 *μ*m, and 63x, scale bar: 20 *μ*m with oil immersion; software: CytoVision. TxR - Texas Red; SpG - Spectrum Green.

**FIGURE 3:**
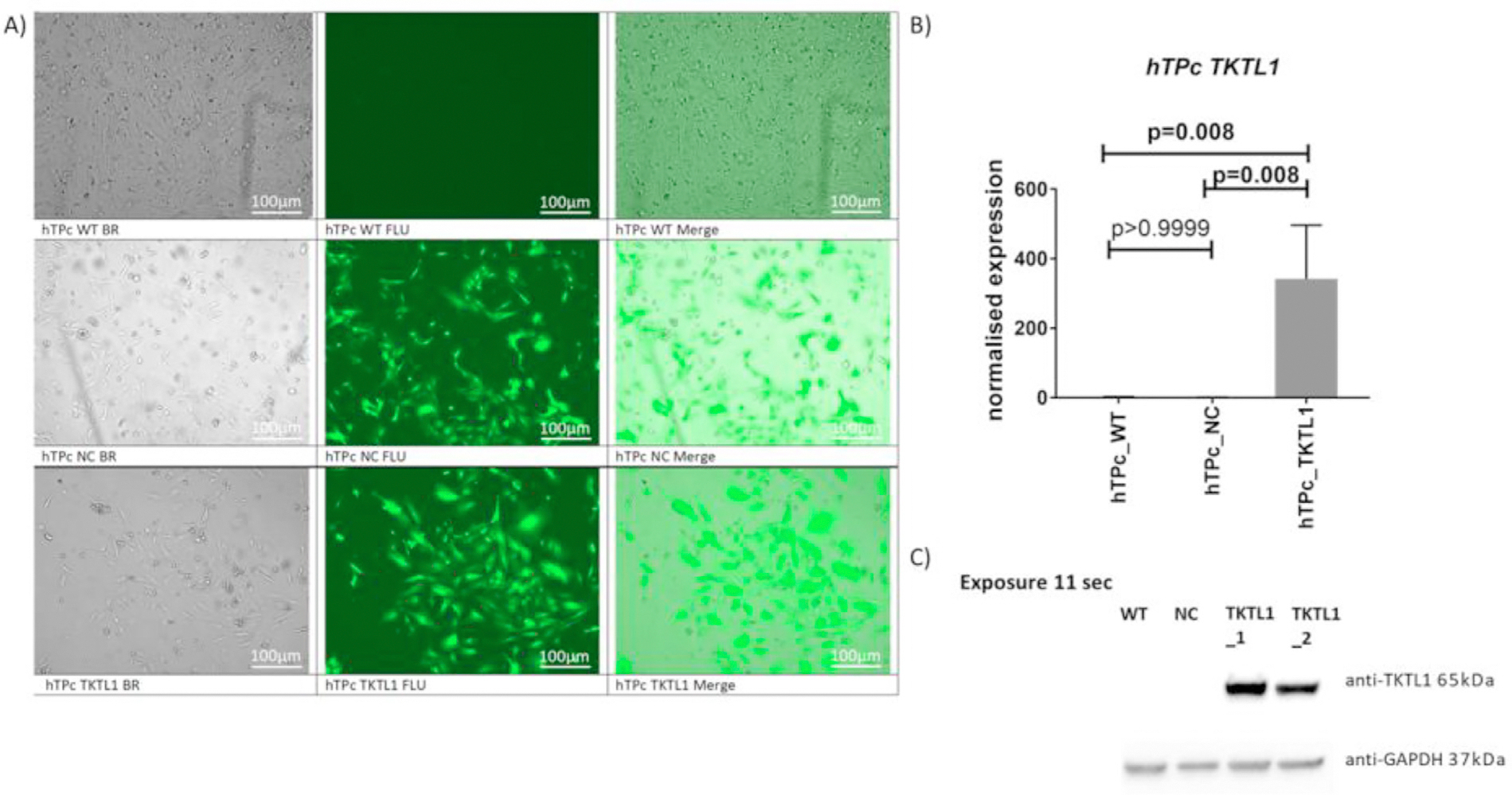
Overexpression of the TKTL1 gene using the lentiviral system. (A) The hTP cells at 48 h after transduction in a Juli FL fluorescence microscope; scale bar: 100 mm. (B) The real-time PCR analysis of TKTL1 gene expression. (C) Representative Western blot analysis of TKTL1 protein, exposure time 11 s; and its graph of the relative quantity of the TKTL1 protein normalized with reference to GAPDH analysed by Image Lab 6.1 tools. Legend: WT – wild type cells in in vitro culture medium only, NC – negative control with GFP expression, TKTL1 – cells with overexpression of TKTL1 gene, BR brightfield, FLU fluorescence.

**FIGURE 4:**
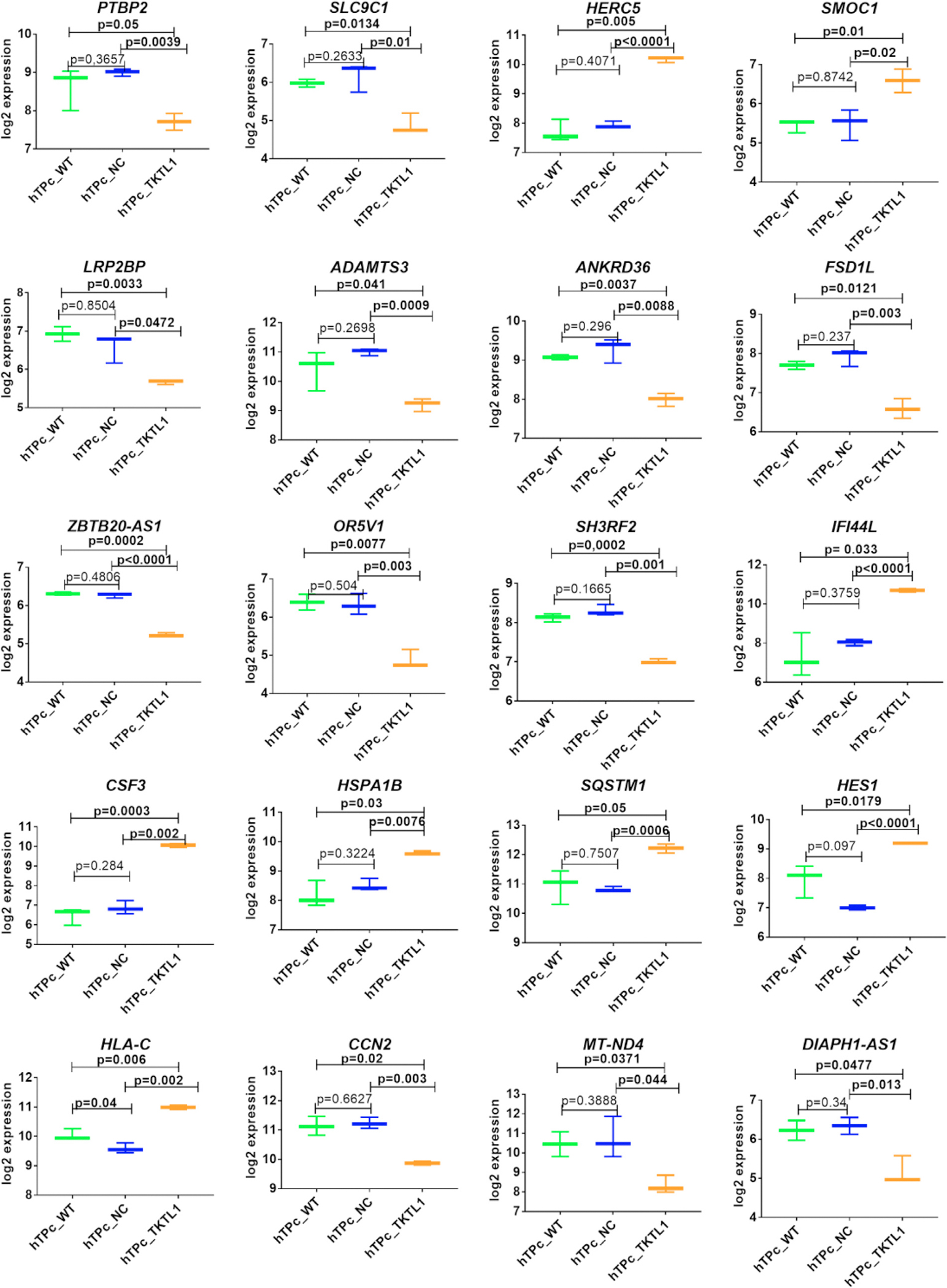
Box-plot graphs of the 20 selected genes assessed in hTP cells with overexpressed TKTL1 gene and controls analysed by RNA-seq. The 8 out of the selected genes were upregulated and 12 downregulated with a minimum of a twofold change (p < 0.05) in hTP cell suspension with overexpression of TKTL1 in comparison to both applied controls. Legend: WT – wild type cells in in vitro culture medium only, NC – negative control with GFP expression, TKTL1 – cells with the overexpressed TKTL1 gene. *p < 0.05, **p < 0.01, ***p < 0.001, ****p < 0.0001.

**FIGURE 5:**
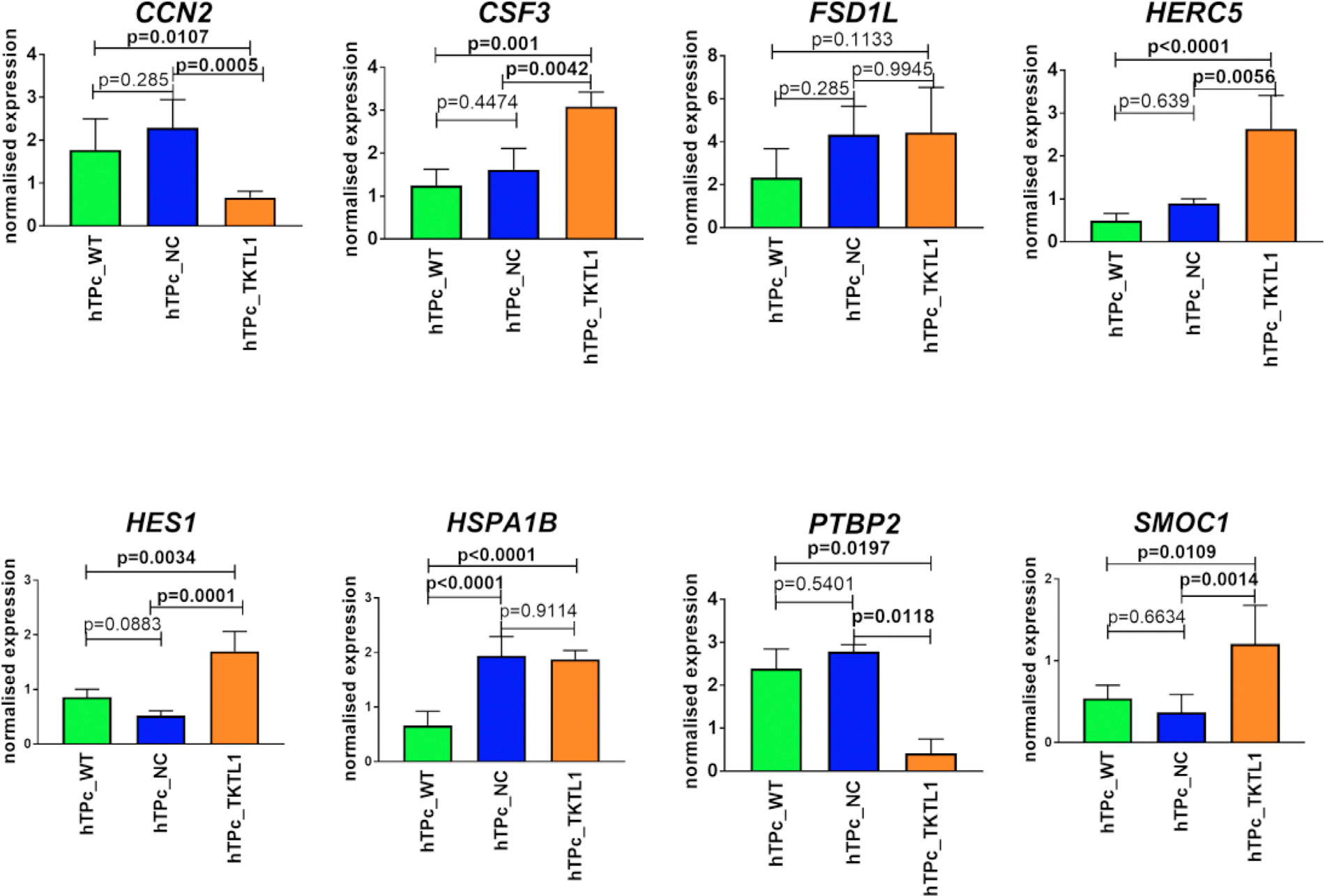
Verification of selected genes by real-time PCR (CCN2, CSF3, FSD1L, HERC5, HES1, HSPA1B, PTBP2, SMOC1) in hTP cells with overexpressed TKTL1 gene versus controls. The CSF3, HERC5, HES1, SMOC1, were upregulated (p < 0.05), and CCN2, PTBP2 were downregulated (p < 0.05) in hTP cells with TKTL1 overexpression as compared to applied controls. Legend: WT – wild type cells in in vitro culture medium only, NC – negative control with GFP expression, TKTL1 – cells with the overexpressed TKTL1 gene. *p < 0.05, **p < 0.01, ***p < 0.001, ****p < 0.0001.

**FIGURE 6:**
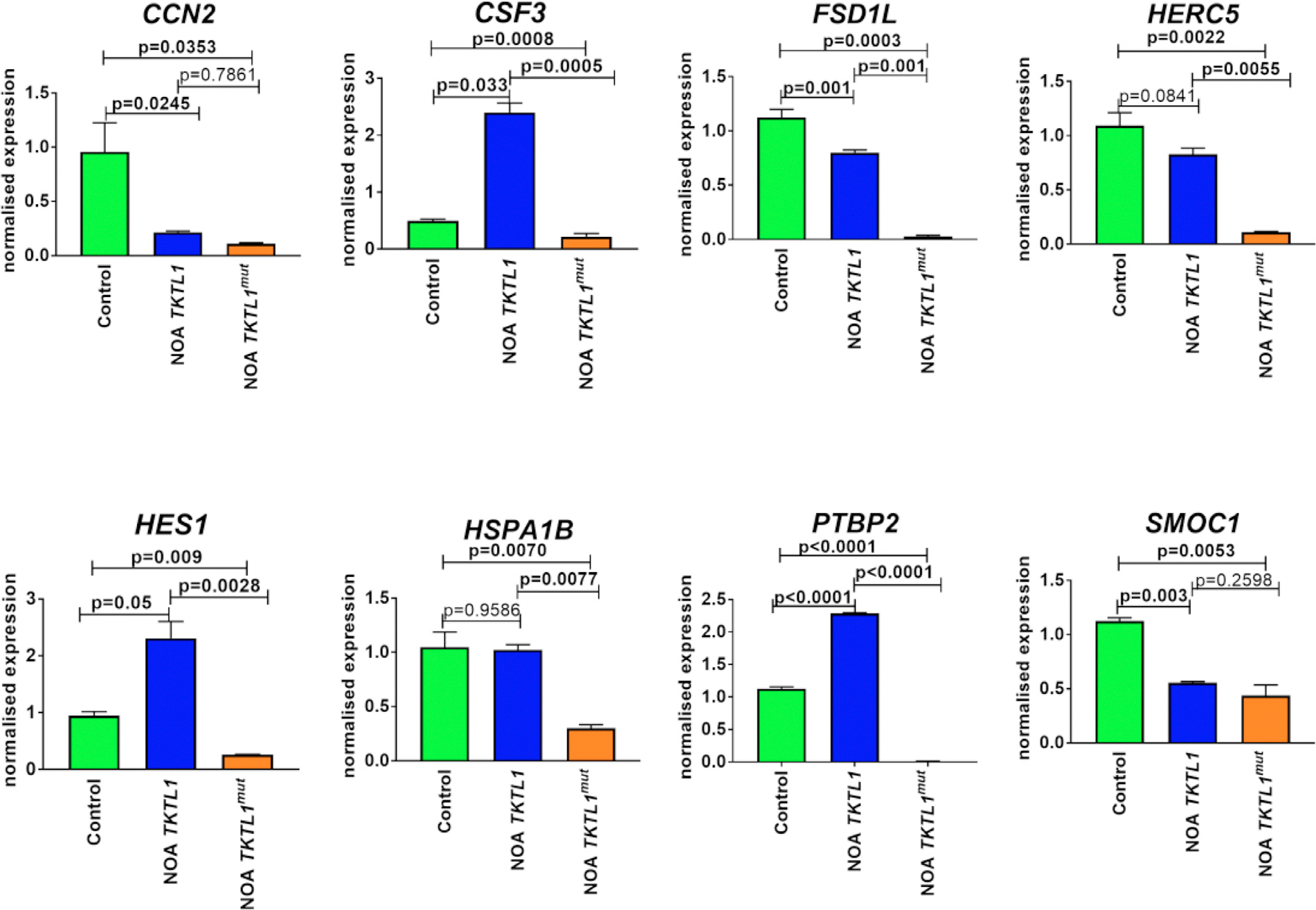
Verification of selected gene by real-time PCR (CCN2, CSF3, FSD1L, HERC5, HES1, HSPA1B, PTBP2, SMOC1) in patients with NOA and controls. The CCN2, CSF3, FSD1L, HERC5, HES1, HSPA1B, PTBP2 genes were downregulated (p < 0.05) in the male gonad tissue sample obtained from an azoospermic patient with a mutation in the TKTL1 gene in comparison to an azoospermic patient without mutation in the TKTL1 gene and control testicular tissue. Legend: Control – male gonadal tissue from men with normal spermatogenesis; AZO without mut. in the TKTL1 gene – patient with non-obstructive azoospermia at meiotic arrest without mutation in the TKTL1 gene; AZO with mut. in the TKTL1 gene – patient with non-obstructive azoospermia at meiotic arrest (34 L) with mutation in the TKTL1 gene. *p < 0.05, **p < 0.01, ***p < 0.001, ****p < 0.0001

