# Supplementary material for: TKTL1: a new candidate gene in non-obstructive azoospermia
Source: Reprod Biomed Online. Author manuscript; Available in PMC 2025 Sep 19. (PMC12448108; doi:10.1016/j.rbmo.2025.104895)
Supplement: MMC1 [file NIHMS2062683-supplement-MMC1.docx]

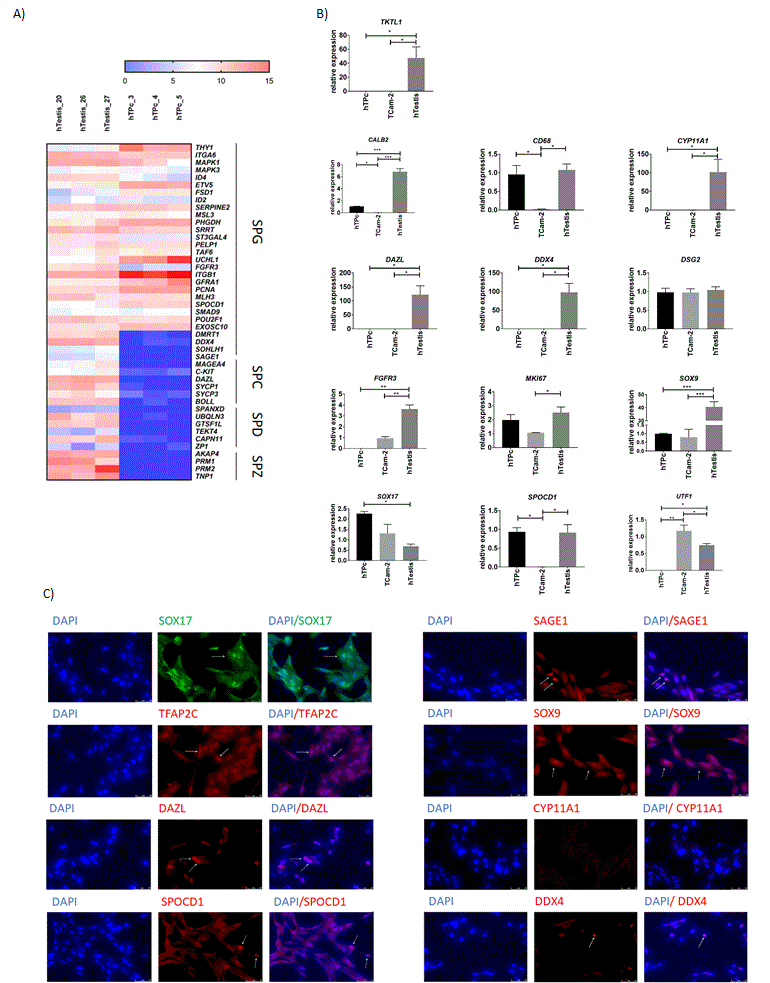


**Supplementary Figure 1.** Gene expression profiles analysis. **A)** Heatmap of spermatogenic markers gene expression in hTP cells in comparison to adult testicular tissue with normal spermatogenesis. **B)** Expression analysis of *CALB2, CD68, CYP11A1, DAZL, DDX4, DSG2, FGFR3, MKI67, SOX9, SOX17, SPOCD1* in the hTPc compared to adult testicular tissue with normal spermatogenesis and Tcam-2 cells. C) Immunofluorescence of SOX17, TFAP2C, DAZL, SPOCD1, SAGE1, CYP11A1, DDX4 protein markers in hTPc. A Leica DMi8 with a proper filter set (DAPI/TxR/Triple) was used; objectives: 40x, scale bar: 50 μm; software: LASX.

Legend: *-p<0.05, **-p<0,01, ***-p<0,001; SPG-spermatogonia, SPC- spermatocytes, SPD- spermatids, SPZ- spermatozoa


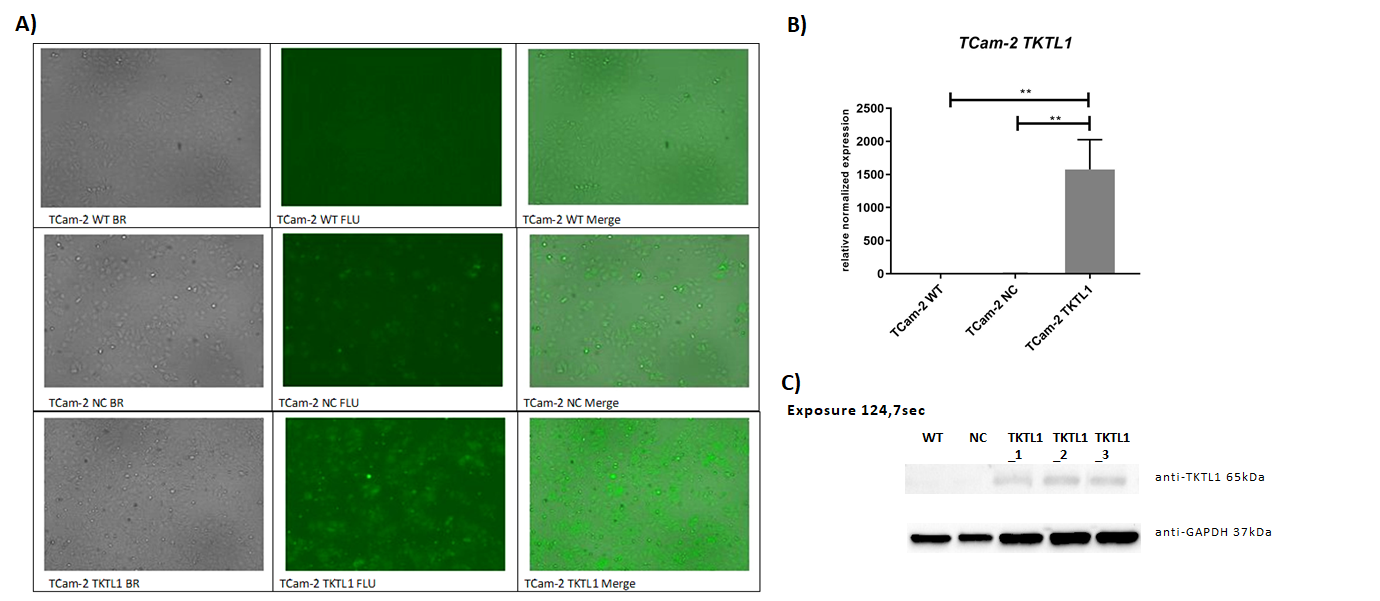


**Supplementary Figure 2.** Activation of the *TKTL1* gene using the CRISPRa system. A) TCam-2 cells at 72 h after transfection under a Juli FL fluorescence microscope; scale bar: 100 μm. B) Real-time PCR analysis of *TKTL1* gene expression. C) Representative Western blot analysis of *TKTL1* protein isolated from TCam-2 cells, exposure time 124,7 sec.

Legend: WT- wild type; NC- negative control with nonspecific sgRNAs for the human genome; TKTL1- cells with the activated *TKTL1* gene using specific sgRNAs for the *TKTL1* sequence; BR- brightfield; FLU- fluorescence


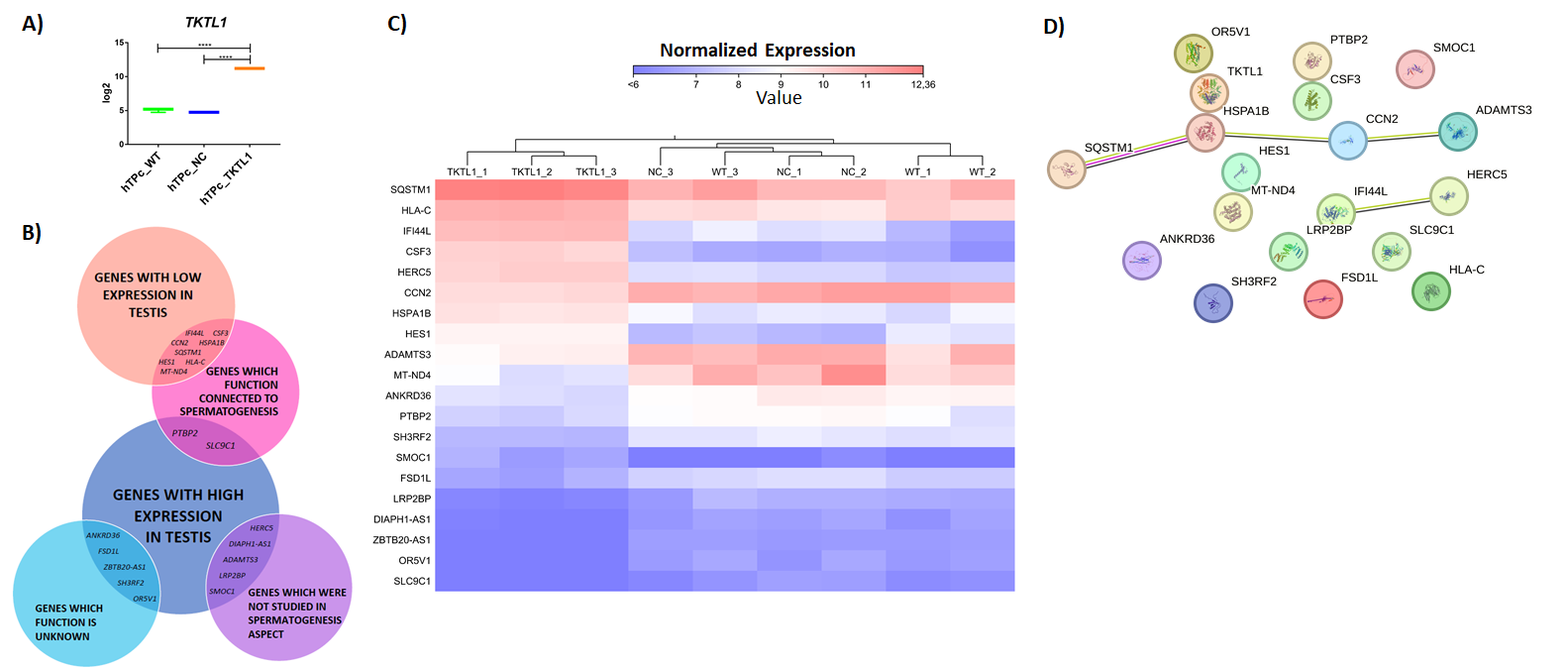


**Supplementary Figure 3.** RNA sequencing (RNA-seq) analysis revealed genes that were differentially expressed following overexpression of the *TKTL1* gene. A) Confirmation of the *TKTL1* overexpression in hTP cells by RNA-seq analysis. B) Venn diagram of the selected genes. C) Heatmap of the 20 selected differentially expressed genes (p<0.05) in *TKTL1*-overexpressed hTP cells and controls.
Legend: WT- wild type; NC- negative control with GFP expression; TKTL1- cells with the overexpression of *TKTL1* gene. D) STRING gene analysis of potential interactions between *TKTL1* and protein products of genes selected from RNA-seq analysis.

**Supplementary Table 1.** The primer sequences used for real-time PCR.

| **Gene symbol** | **Primer** | **Primer sequence (5’🡪3’)** | **Product length** |
| --- | --- | --- | --- |
| *TKTL1* | Forward | CTGTGGGGTATCTGTTGGTGA | 212 bp |
|  | Reverse | AGCGTTCTTGTGGGGTGTAA |  |
| *ACTB* | Forward | CTTCCTGGGCATGGAGTCC | 192 bp |
|  | Reverse | ATCTTGATCTTCATTGTGCTG |  |
| *CCN2* | Forward | CTGGAAGAGAACATTAAGAAGGGCA | 244 bp |
|  | Reverse | AATGGCAGGCACAGGTCTTG |  |
| *CSF3* | Forward | AGCCCCACCCAGACCC | 226 bp |
|  | Reverse | GGCACACTCACTCACCAGC |  |
| *FSD1L* | Forward | ACCATCCCCAAAACGAACATCT | 109 bp |
|  | Reverse | TTCAATAGCAGTGTCTCCCAGC |  |
| *HERC5* | Forward | GGAAAATGACTGTGGACGCT | 163 bp |
|  | Reverse | ATTGCTGCCGACCTAAGATAAG |  |
| *HES1* | Forward | GCTCTGAAGAAAGATAGCTCGC | 212 bp |
|  | Reverse | TCGGTATTAACGCCCTCGC |  |
| *HSPA1B* | Forward | GTGTGTAACCCCATCATCAGC | 169 bp |
|  | Reverse | CCCAACAGTCCACCTCAAAGA |  |
| *PTBP2* | Forward | CTCTTAGGGCTTCCTGTTGC | 177 bp |
|  | Reverse | TTGGGGCGTAACCATCTCTT |  |
| *SMOC1* | Forward | CCAGGTGCTGTGCCTATTCA | 258 bp |
|  | Reverse | CCCGAGAAGGTTGTGCTCC |  |
| *CALB2* | Forward | TTACGACAAGGATAGAAGCGG | 231 bp |
|  | Reverse | GGGGAGGTGGAGAAGCAG |  |
| *CD68* | Forward | CACACAGGGGTCTTTGGGCA | 144 bp |
|  | Reverse | GTAGGCGGATGGGCGTCTC |  |
| *CYP11A1* | Forward | CCAAGGGTCTTCCCCCA | 297 bp |
|  | Reverse | TGACATAAACCGACTCCACG |  |
| *DDX4* | Forward | GATACCAGAAAGGGCAAGAGCA | 213 bp |
|  | Reverse | GGAGTGAGAATACAAGGACAGGAG |  |
| *DSG2* | Forward | GCCTGCTTATCCTCCAGTGTTCT | 90 bp |
|  | Reverse | CTGCTGTGTTCCTCTCTGTCCAA |  |
| *FGFR3* | Forward | CTGTCTGGGTCAAGGATGGC | 179 bp |
|  | Reverse | CGAGGATGGAGCGTCTGTC |  |
| *GAPDH* | Forward | GCTCTCTGCTCCTCCTGTTC | 112 bp |
|  | Reverse | ACCAAATCCGTTGACTCCGA |  |
| *MKI67* | Forward | TGCTTGTTTGGAAGGGGTATTGA | 132 bp |
|  | Reverse | TTGTGTTGGATTTGTGGAACTGAAA |  |
| *SOX9* | Forward | ACGCACATCTCCCCCAAC | 173 bp |
|  | Reverse | TTCAGGTCAGCCTTGCCC |  |
| *SOX17* | Forward | GAGCCAAGGGCGAGTCCCGTA | 141 bp |
|  | Reverse | CCTTCCACGACTTGCCCAGCA |  |
| *SPOCD1* | Forward | ACAAGGGCGAAGTGGAGATT | 278 bp |
|  | Reverse | TGGAGCAGGCATAGGAGTTT |  |
| *UTF1* | Forward | GGCACCTGGGCGACATC | 208 bp |
|  | Reverse | CCTGGAGAGGGGAGACTGG |  |

Legand: *TKTL1-* transketolase like 1*, ACTB-* actin beta*, CCN2-* cellular communication network factor 2*, CSF3-* colony stimulating factor 3*, FSD1L-* fibronectin type III and SPRY domain containing 1 like*, HERC5-* HECT and RLD domain containing E3 ubiquitin protein ligase 5*, HES1-* hes family bHLH transcription factor 1*, HSPA1B-* heat shock protein family A (Hsp70) member 1B*, PTBP2-* polypyrimidine tract binding protein 2*, SMOC1-* SPARC related modular calcium binding 1*, CALB2-* calbindin 2*, CD68-* CD68 molecule*, CYP11A1-* cytochrome P450 family 11 subfamily A member 1*, DDX4-* DEAD-box helicase 4*, DSG2-* desmoglein 2*, FGFR3-* fibroblast growth factor receptor 3*, GAPDH-* glyceraldehyde-3-phosphate dehydrogenase*, MKI67-* marker of proliferation Ki-67*, SOX9-* SRY-box transcription factor 9*, SOX17-* SRY-box transcription factor 17*, SPOCD1-* SPOC domain containing 1*, UTF1-* undifferentiated embryonic cell transcription factor 1.
